# From synapse to system: mechanistic pathways of neural signaling dysfunction in psychiatric disorders

**DOI:** 10.3389/fcell.2026.1762930

**Published:** 2026-02-20

**Authors:** Rohan Gupta, Niraj Kumar Jha, Naveen Kumar, Rupak Nagraik, Karthikeyan Ravi

**Affiliations:** 1 Department of Biotechnology and Bioengineering, School of Biosciences and Technology, Galgotias University, Greater Noida, Uttar Pradesh, India; 2 Department of Physics, School of Basic and Applied Sciences, Galgotias University, Greater Noida, Uttar Pradesh, India; 3 Department of Biotechnology, Graphic Era Deemed to be University, Dehradun, Uttarakhand, India; 4 Centre for Herbal Pharmacology and Environmental Sustainability, Chettinad Hospital and Research Institute, Chettinad Academy of Research and Education, Chennai, Tamil Nadu, India

**Keywords:** computational neuroscience, network dysconnectivity, neural signaling, precision psychiatry, psychiatric disorders

## Abstract

Psychiatric disorders are increasingly viewed as network-level brain diseases resulting from disruptions in neural signaling across various hierarchies, including molecular, synaptic, circuit, and systems levels. Evidence indicates that receptor dysregulation, abnormal intracellular pathways, and changes in ion channel activity lead to widespread network dysconnectivity, resulting in cognitive, emotional, and behavioral deficits. This review integrates advancements in genomics, transcriptomics, connectomics, and computational modeling to establish a framework for understanding signaling abnormalities in major psychiatric disorders. Further, this study investigates essential molecular and cellular processes such as synaptic plasticity, receptor-mediated communication, intracellular signaling cascades, and neuroimmune interactions, and connects these to disturbances in oscillatory dynamics, circuit architecture, and overall brain network organization. Additionally, neuroimaging and graph-theoretic studies consistently demonstrate an excitation–inhibition imbalance, atypical synaptic pruning, impaired oscillatory synchrony, and maladaptive connectivity within networks, including the default mode, salience, and fronto-limbic systems, across schizophrenia, depression, bipolar disorder, anxiety, and autism spectrum disorders. Moreover, genetic and epigenetic variations in signaling genes, such as CACNA1C, GRIN2B, and DISC1, along with developmental and environmental factors, contribute to network vulnerability and clinical heterogeneity. Emerging artificial intelligence and multimodal integration methods facilitate the identification of individualized “signaling fingerprints,” which connect molecular perturbations to systems-level dysfunction. This research enhances precision psychiatry and guides targeted interventions based on neuromodulation, molecular mechanisms, and biomarkers.

## Graphical Abstract

**Figure:**
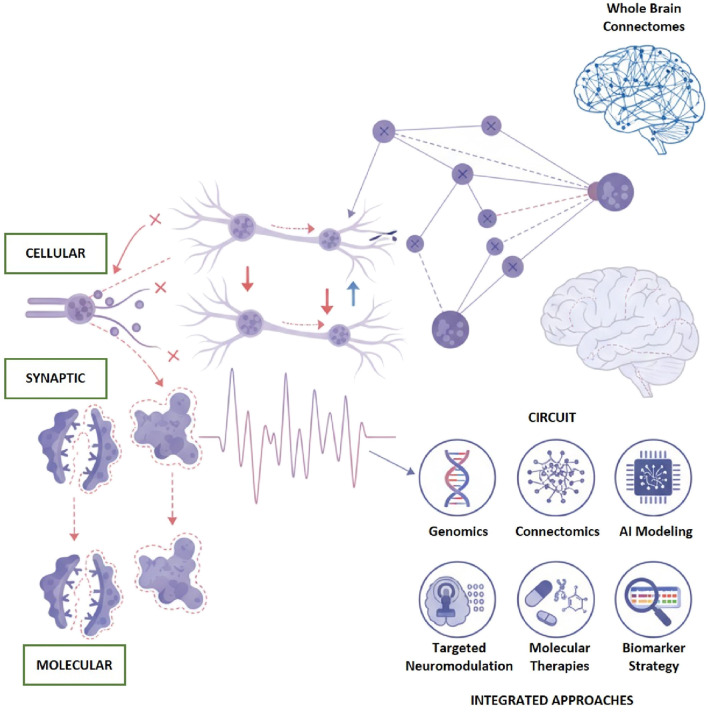
Multilevel disturbances in neuropsychiatric diseases encompass molecular, synaptic, and cellular levels, resulting in circuit-level dysconnectivity and modified whole-brain connectomes. Impaired neurotransmission, abnormal synaptic signaling, and impaired neural communication lead to functional network imbalance. Further, integrated methodologies that merge genomes, connectomics, and AI modeling with targeted neuromodulation, molecular treatments, and biomarker methods seek to connect molecular dysfunctions with a systems-level comprehension and inform precision interventions in neuropsychiatric disorders.

## Highlights

1. Psychiatric disorders result from disruptions in neural signaling across multiple scales.
2. Integrates genomics, connectomics, and computational modeling to provide mechanistic insights.
3. Identifies excitation-inhibition imbalance and network dysconnectivity as common mechanisms.
4. Identifies signaling fingerprints that connect molecular dysfunctions to associated symptoms.
5. Proposes interventions based on precision neuromodulation and AI-driven biomarkers.

## 1 Introduction

**FIGURE 1:**
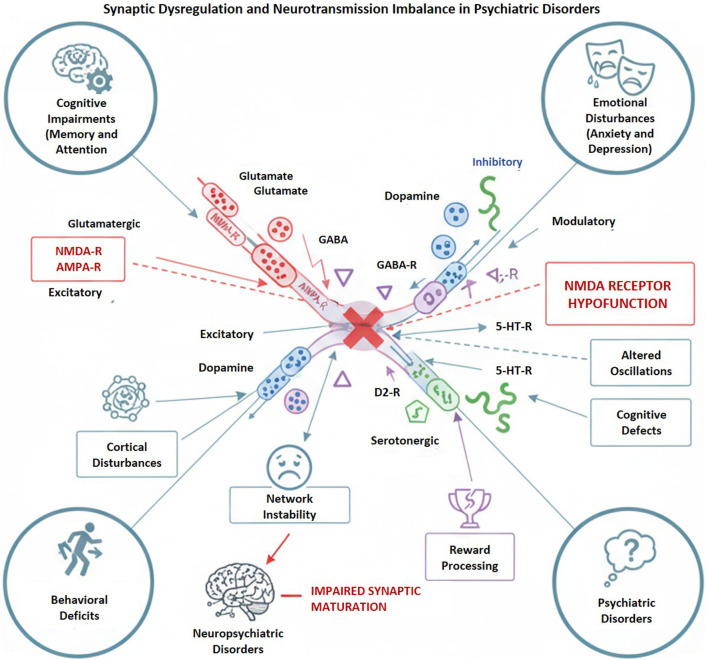
Synaptic dysregulation and neurotransmission imbalance in neuropsychiatric diseases: Modified excitatory (glutamatergic), inhibitory (GABAergic), and modulatory (dopaminergic, serotonergic) transmission, especially NMDA receptor hypofunction, undermines network stability and synaptic development. These alterations result in cortical disruptions, cognitive impairments, emotional dysregulation, and behavioral abnormalities that underpin the pathophysiology of neuropsychiatric and psychiatric disorders.

Psychiatric disorders represent a diverse range of brain diseases that extend beyond the conventional notion of isolated neurotransmitter imbalances, reflecting widespread disruptions in neural communication across multiple biological levels. Evidence from molecular neurobiology, systems neuroscience, and computational psychiatry suggests that these disorders result from abnormal neural signaling processes that hinder the brain’s capacity to transmit, process, and integrate information effectively (Insel and Cuthbert, 2015). Additionally, psychiatric pathophysiology encompasses complex interactions among molecular, synaptic, circuit, and network-level dysfunctions, rather than being limited to disturbances in monoaminergic transmission, such as deficiencies in serotonin or dopamine (Friston, 2023). Neural signaling at the molecular level is regulated by interactions between receptors and transporters, intracellular signaling cascades, and the regulation of synaptic proteins, all of which collectively affect neurotransmitter release, uptake, and receptor responsiveness. Further, dysregulation at this level results in subsequent impairments in synaptic plasticity, excitability, and synaptogenesis, consequently affecting information encoding and adaptive learning (Martella, 2023). Mounting evidence have shown that disturbances in neural oscillations and impaired synchrony within and between brain regions, especially in thalamo-cortical and cortico-limbic pathways, compromise the temporal coordination essential for cognition and emotion. These abnormalities manifest at the systems level, where disruptions in the integration of large-scale networks, particularly the default mode, salience, and central executive networks, lead to the cognitive, affective, and behavioral disturbances typical of psychiatric disorders (Gonzalez-Escamilla et al., 2023). These multi-scale alterations collectively establish a common mechanistic substrate across various conditions, including schizophrenia (SCZ), major depressive disorder (MDD), bipolar disorder, anxiety, post-traumatic stress disorder (PTSD), and autism spectrum disorders (ASD) (Rootes-Murdy et al., 2024). These disorders, despite their diverse clinical manifestations, share underlying neural principles, such as excitation-inhibition imbalance, deficits in synaptic signaling, and dysconnectivity at the network level (Sun H. et al., 2025). This indicates that psychiatric symptoms arise from dynamic failures in neural coordination rather than from localized neurotransmitter deficiencies. This review seeks to connect molecular neurobiology with network neuroscience by synthesizing findings from genomics, connectomics, and computational modeling to clarify how signaling abnormalities disseminate through various hierarchical levels of brain organization. The following review emphasizes advancements in neuromodulation, pharmacological innovation, and artificial intelligence (AI)-driven modeling that utilize mechanistic insights to enhance precision psychiatry. Lastly, this framework aims to connect molecular perturbations with large-scale network dysfunction, thereby offering a comprehensive understanding of psychiatric disorders and informing the creation of more targeted, biologically based therapeutic strategies (Figure 1).

## 2 Molecular and cellular signaling aberrations

**FIGURE 2:**
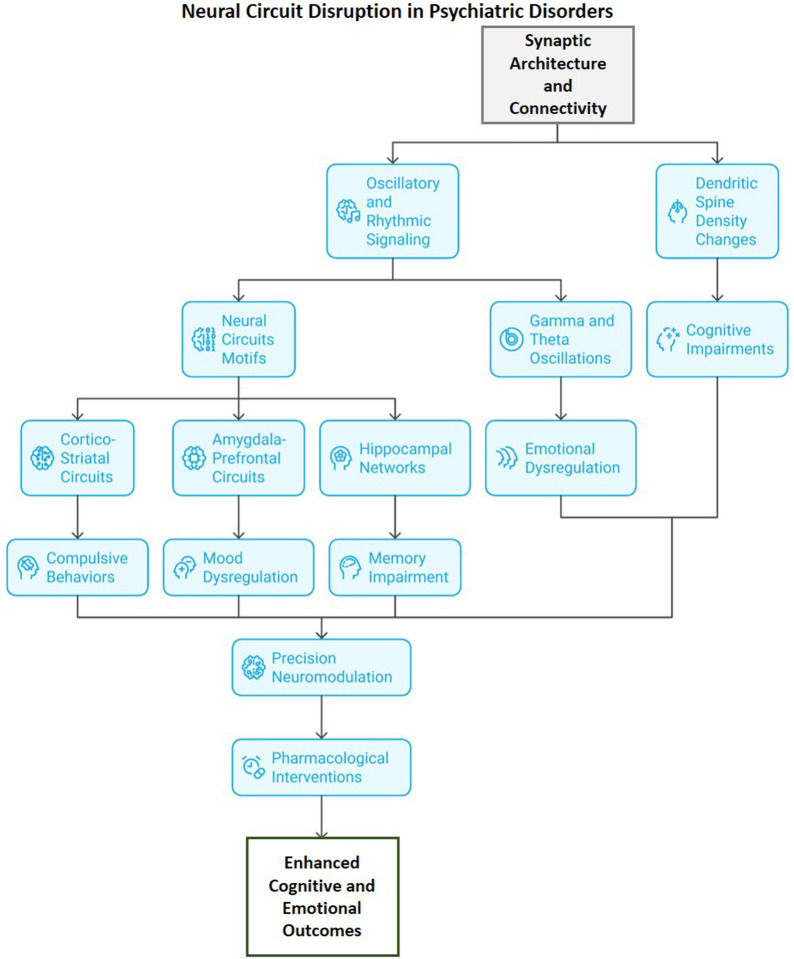
Disruption of neural circuits in mental diseases: Modifications in synaptic structure and connectivity result in disrupted oscillatory signaling and alterations in dendritic spines, compromising network motifs including cortico-striatal, amygdalo-prefrontal, and hippocampal circuits. These disturbances lead to obsessive behaviors, mood dysregulation, emotional instability, and memory impairments. Targeted precision neuromodulation and pharmaceutical treatments seek to restore circuit integrity, hence improving cognitive and affective outcomes in neuropsychiatric disorders.

Dysregulated synaptic transmission and plasticity play a crucial role in psychiatric pathophysiology, disturbing the equilibrium between excitatory and inhibitory neurotransmission within glutamatergic, GABAergic, dopaminergic, and serotonergic systems. Hypofunction of NMDA receptors results in cortical disinhibition, disrupted oscillations, and cognitive impairments in SCZ, whereas abnormal NMDA-mediated signaling influences synaptic maturation in autism (Dwyer et al., 2024; Tumdam et al., 2024). GABAergic and monoaminergic dysfunctions additionally compromise network stability, reward processing, and mood regulation. These alterations collectively impair synaptic plasticity, leading to cognitive, emotional, and behavioral deficits typical of psychiatric disorders (Cutler et al., 2023) (Table 1) (Figure 2). Further, at the molecular and cellular level, several alterations emerge as putative upstream drivers of circuit dysfunction rather than downstream correlates. These include NMDA receptor hypofunction, impaired GABAergic interneuron signaling, epigenetic repression of synaptic plasticity genes, and chronic neuroimmune activation. Experimental manipulation of these factors causally alters synaptic gain and excitation-inhibition balance, particularly within prefrontal–hippocampal and thalamo-cortical circuits (Yizhar et al., 2011; Lewis et al., 2012; Anticevic et al., 2015). For example, NMDA receptor hypofunction preferentially disrupts parvalbumin-positive interneurons, reducing inhibitory control over pyramidal neurons and leading to aberrant cortical excitability. Similarly, HDAC2 or HDAC3-mediated chromatin repression decreases expression of synaptic scaffolding and plasticity-related genes, weakening long-range circuit integration (Homayoun and Moghaddam, 2007; Guan et al., 2009; McQuown et al., 2011). These molecular perturbations therefore represent upstream nodes through which genetic, metabolic, and inflammatory stressors converge to destabilize defined circuit motifs.

**TABLE 1:** Summary of key signaling pathways and their associated psychiatric phenotypes.

Signaling molecule/Pathway	Primary pathway type	Experimental/Model evidence	Psychiatric phenotypes associated	Mechanistic outcomes	Key findings/Insights	Limitations/Drawbacks
NMDA receptor (GRIN2B-mediated)	Glutamatergic signaling	NMDA receptor antagonists (ketamine, PCP) models; postmortem cortical tissue	SCZ, ASD	Cortical disinhibition, impaired gamma oscillations, cognitive deficits	NMDA hypofunction leads to E/I imbalance and network desynchronization	Translational gap between animal models and human cognitive symptoms
GABAergic system (GAD1, PV^+^ interneurons)	Inhibitory neurotransmission	PV^+^ interneuron-deficient mice; EEG/fMRI coherence studies	SCZ, MDD, anxiety	Reduced inhibitory tone, network instability, abnormal oscillations	GABAergic dysfunction disrupts cortical synchrony and working memory	Heterogeneous GABA alterations across brain regions
Dopaminergic (D2 receptor, mesocorticolimbic)	Monoaminergic modulation	PET/fMRI studies; pharmacological modulation (antipsychotics)	SCZ, bipolar disorder, addiction	Dysregulated reward processing and salience attribution	Hyperdopaminergia in striatum, hypodopaminergia in PFC	Oversimplified dopaminergic hypotheses overlook network effects
Serotonergic (5-HT1A, 5-HT2A)	Monoaminergic/receptor signaling	Chronic stress models; receptor binding assays	Depression, anxiety	Mood dysregulation, stress reactivity	Serotonin receptor modulation impacts affective regulation	Delayed therapeutic onset; receptor subtype complexity
cAMP–PKA–CREB	Intracellular second messenger	CREB knockouts; antidepressant exposure models	Depression, SCZ	Impaired transcriptional regulation, anhedonia	CREB activation critical for plasticity and mood regulation	Difficult to isolate from other convergent cascades
MAPK/ERK	Neurotrophic and synaptic plasticity	Stress-induced signaling models; postmortem cortical data	Depression, bipolar disorder	Altered dendritic remodeling, maladaptive neuroplasticity	Hyperactivation correlates with impaired emotion regulation	Highly context-dependent; bidirectional effects
PI3K–Akt–mTOR	Growth and metabolic signaling	Rodent models; transcriptomic enrichment in patient tissue	Bipolar disorder, ASD, SCZ	Reduced synaptic protein synthesis and neurotrophic support	Pathway regulates resilience and energy metabolism	mTOR modulation shows inconsistent behavioral outcomes
BDNF–TrkB	Neurotrophic signaling	BDNF knockout mice; serum/plasma BDNF levels	Depression, SCZ, PTSD	Impaired synaptic growth, mood dysregulation	Reduced BDNF linked with stress-induced atrophy	Peripheral BDNF may not reflect CNS levels
Wnt/β-catenin	Developmental/neuroplastic signaling	iPSC-derived neurons; GWAS and methylation studies	SCZ, ASD	Aberrant synaptogenesis, dendritic instability	Key in neurodevelopmental vulnerability	Functional redundancy across Wnt ligands
Voltage-gated Ca^2+^ channels (CACNA1C, CACNA1H)	Electrophysiological signaling	Genetic association; Ca^2+^ imaging in patient-derived neurons	Bipolar disorder, SCZ	Disrupted calcium homeostasis, abnormal excitability	Links intracellular signaling to affective instability	Functional validation across cell types limited
K^+^ channel (KCNH2, KCNN3)	Ion channel/excitability regulation	Patch-clamp electrophysiology; genetic variants	SCZ, mood disorders	Neuronal hyperexcitability, disrupted rhythmicity	Channelopathies cause circuit desynchronization	Species differences complicate translation
Neuroimmune (IL-6, TNF-α, microglial activation)	Inflammatory/cytokine signaling	Human plasma cytokine assays; microglia activation imaging	Depression, SCZ	Synaptic pruning, neuroinflammation, oxidative stress	Chronic inflammation alters neurotransmission and plasticity	Difficult to distinguish cause from consequence
Mitochondrial–AMPK–PGC1α	Metabolic/oxidative signaling	Postmortem tissue; mitochondrial function assays	Bipolar disorder, MDD, SCZ	Energy failure, oxidative stress, reduced resilience	Links bioenergetic imbalance to cognitive dysfunction	Mitochondrial heterogeneity across cell types
Complement–C4A mediated pruning	Synaptic immune signaling	C4A overexpression models; GWAS	SCZ	Excessive synaptic elimination, cortical thinning	Strong genetic and mechanistic link to SCZ	Limited temporal mapping in humans
DISC1/cAMP-PKA/PI3K-Akt interaction	Developmental/intracellular scaffolding	DISC1 mutant mice; human iPSC neurons	SCZ, MDD	Abnormal neuronal migration and synaptic organization	Connects polygenic risk to circuit disorganization	Complex protein–protein interaction network
Epigenetic regulators (BDNF, GAD1 methylation; miR-137)	Epigenetic/transcriptional control	DNA methylation assays; miRNA transcriptomics	SCZ, MDD, bipolar	Altered gene expression, impaired plasticity	Environment-driven transcriptional repression of plasticity genes	Causality vs. consequence unclear
Thalamo-cortical oscillatory synchronization	Systems/rhythmic signaling	EEG/fMRI coherence; TMS perturbation models	SCZ, depression, anxiety	Abnormal gamma/theta coupling, impaired cognition	Oscillatory deficits link microcircuit and network dysfunction	Signal interpretation confounded by comorbidities

### 2.1 Synaptic transmission and plasticity dysregulation

Aberrant synaptic transmission and maladaptive plasticity are fundamental mechanisms that underlie the pathophysiology of psychiatric disorders. The balance between excitatory and inhibitory neurotransmission is crucial for the stability of neural networks, cognitive flexibility, and emotional regulation. The disruption of this equilibrium, particularly within glutamatergic, GABAergic, dopaminergic, and serotonergic systems, has been implicated in various psychiatric conditions, including SCZ, ASD, depression, and bipolar disorder (Zhang J. et al., 2024). Further, glutamatergic signaling, primarily mediated by N-methyl-D-aspartate (NMDA) and α-amino-3-hydroxy-5-methyl-4-isoxazolepropionic acid (AMPA) receptors, regulates synaptic strength, learning, and memory (Stockwell et al., 2024). In SCZ, the hypofunction of NMDA receptors has become a central hypothesis, resulting in cortical disinhibition, impaired gamma oscillations, and cognitive deficits. Studies have shown that the hypofunction leads to a decrease in excitatory input to inhibitory interneurons, particularly parvalbumin-positive (PV) cells, causing an imbalance between excitation and inhibition at the network level and resulting in abnormal cortical synchrony (Roach et al., 2025). In ASD, deficits in NMDA receptor-mediated signaling lead to abnormal synaptic maturation and altered sensory processing. Additionally, excessive excitation or insufficient inhibition during critical developmental periods may disrupt functional connectivity. GABAergic dysfunction exacerbates this imbalance, as impaired inhibitory tone compromises network oscillations critical for attention and working memory (Jiang et al., 2022; Zhao et al., 2022; Tumdam et al., 2024). Concurrent disruptions in dopaminergic and serotonergic neurotransmission influence reward processing, mood, and social behavior, resulting in shared neurochemical foundations among various disorders. Further, dopamine dysregulation within mesocorticolimbic circuits plays a role in both the positive and negative symptoms of SCZ, while serotonergic abnormalities affect affective stability and stress reactivity in mood disorders (Hahn et al., 2025; Peng et al., 2025). In addition to receptor-level dysfunction, disruptions in synaptic plasticity, such as long-term potentiation (LTP) and long-term depression (LTD), hinder adaptive learning and circuit remodeling. These changes in neurotransmitter signaling and synaptic dynamics indicate a failure in the brain’s capacity to optimize information processing, resulting in the cognitive, emotional, and behavioral disruptions typical of psychiatric disorders (Appelbaum et al., 2023; Mäki-Marttunen et al., 2024). Comprehending these interconnected systems provides essential insights for targeted interventions that aim to restore synaptic balance and enhance functional neural plasticity.

### 2.2 Intracellular signaling pathways

The dysregulation of intracellular signaling pathways is a significant molecular mechanism involved in the pathophysiology of psychiatric disorders. These pathways function as integrative hubs that convert extracellular neurotransmitter activity into enduring changes in gene expression, synaptic plasticity, and neuronal survival (Chen Y. et al., 2024). Prominent signaling cascades include cAMP-PKA-CREB, mitogen-activated protein kinase/extracellular signal-regulated kinases (MAPK/ERK), phosphoinositide 3-kinase-protein kinase B (PI3K-Akt), mammalian target of rapamycin (mTOR), and Wnt, each contributing uniquely and interdependently to neuronal homeostasis and adaptive responses (Lima Caldeira et al., 2019). The cAMP-PKA-CREB pathway modulates transcriptional programs that are critical for synaptic plasticity, learning, and mood regulation. For example, impaired CREB activation is linked to cognitive deficits, anhedonia, and changes in stress responsiveness, especially in the context of depression and SCZ (Balu and Coyle, 2018; Sahay et al., 2024b). Likewise, the MAPK/ERK cascade, responsible for activity-dependent synaptic remodeling and dendritic spine dynamics, exhibits abnormal activation patterns in mood and psychotic disorders, resulting in maladaptive neuroplastic changes (Li et al., 2023). The PI3K-Akt-mTOR pathway regulates protein synthesis and cellular metabolism, connecting intracellular energy balance with synaptic growth and resilience. Additionally, the dysregulation of this pathway leads to compromised neurotrophic signaling and cellular stress responses, as evidenced in bipolar disorder and ASD (Vanderplow et al., 2021; Chen Y. et al., 2024). Moreover, Studies have shown that the Wnt signaling pathway, essential for neuronal differentiation and synapse formation, shows altered expression and downstream signaling in SCZ, indicating its role in neurodevelopmental vulnerability (Sahay et al., 2024a). A significant aspect of these cascades is their interaction with neurotrophic signaling, especially the brain-derived neurotrophic factor-tropomyosin receptor kinase B (BDNF-TrkB) pathway, which is crucial for neuronal growth, survival, and synaptic strength. Decreased BDNF expression or compromised TrkB receptor activation disrupts downstream CREB and Akt signaling, resulting in synaptic destabilization and mood dysregulation. These intracellular signaling networks function as a precise molecular interface linking environmental stimuli, neurotransmitter systems, and genomic regulation (Fan et al., 2025). Furthermore, dysregulation affects neuronal adaptability, leading to persistent structural and functional abnormalities in psychiatric disorders. This presents potential targets for molecular and pharmacological interventions designed to restore signaling homeostasis and improve neuroplasticity.

### 2.3 Ion channels and electrophysiological dynamics

Ion channels are crucial in regulating neuronal excitability, synaptic transmission, and network synchronization, which are processes vital for cognition, emotion, and behavior. Studies have demonstrated that ion channel dysfunction, referred to as channelopathies, has been increasingly associated with the pathophysiology of psychiatric disorders, especially those marked by mood instability, psychosis, and cognitive impairment (Imbrici et al., 2013). Further, voltage-gated sodium (Na^+^), potassium (K^+^), calcium (Ca^2+^), and chloride (Cl^−^) channels are essential for the generation and propagation of action potentials, as well as for maintaining the excitatory-inhibitory balance in neural circuits. Alterations in genetic and functional aspects of these channels can impair neuronal firing precision, synaptic integration, and oscillatory synchrony, resulting in extensive network instability (Palmisano V. F. et al., 2024; Qiu et al., 2025). Additionally, mutations in voltage-gated Ca^2+^ channels, such as *CACNA1C* and *CACNA1H*, have been significantly linked to bipolar disorder and SCZ, indicating a connection between altered intracellular calcium signaling, dysregulated neurotransmitter release, and impaired synaptic plasticity (Allen et al., 2024; Han, 2025). Moreover, abnormalities in K^+^ channel genes, including *KCNQ2*, *KCNH2*, and *KCNN3*, are associated with hyperexcitability, mood dysregulation, and heightened vulnerability to psychosis due to their effects on neuronal repolarization and rhythmic activity. However, variants in Na^+^ and Cl^−^ channels contribute to disrupted excitability and altered gamma oscillations, which are neural signatures linked to deficits in working memory and perceptual coherence in SCZ (Alam et al., 2023; Nakanishi et al., 2023). These channelopathies collectively disrupt the temporal coordination of neural networks, leading to abnormal signaling patterns that contribute to fundamental psychiatric symptoms (Verriello et al., 2025). Thus, examining the role of specific ion channel dysfunctions in altering electrophysiological dynamics offers essential insights into psychiatric pathophysiology and reveals potential therapeutic avenues for reestablishing circuit stability and cognitive-emotional regulation.

### 2.4 Neuroimmune and inflammatory modulation

Neuroimmune signaling and chronic low-grade inflammation are significant factors influencing brain function and the pathophysiology of major psychiatric disorders, especially depression and SCZ. The central nervous system (CNS), previously deemed immunoprivileged, is now acknowledged as an active immunological organ (Nusslock et al., 2024; Feng et al., 2025). In the CNS, complex interactions among neurons, glia, and peripheral immune mediators influence neural signaling and behavior. For instance, dysregulated cytokine signaling, microglial activation, and neuroinflammatory feedback loops contribute to changes in synaptic plasticity, neurotransmission, and network connectivity (Tastan and Heneka, 2024). A study was conducted in subsets of patients with MDD and SCZ, where elevated levels of proinflammatory cytokines, including interleukin-1β (IL-1β), interleukin-6 (IL-6), and tumor necrosis factor-alpha (TNF-α), are consistently observed (Tastan and Heneka, 2024). Additionally, cytokines either traverse the blood-brain barrier (BBB) or are synthesized by resident immune cells, thereby modulating neurotransmitter synthesis, especially serotonin, dopamine, and glutamate, and affecting receptor sensitivity and synaptic vesicle dynamics (Nava et al., 2025; Nayak et al., 2025). Moreover, chronic inflammatory signaling activates microglia, resulting in excessive synaptic pruning, oxidative stress, and disruption of neurotrophic pathways like BDNF-TrkB, which impairs neuronal survival and connectivity. In SCZ, abnormal microglial-mediated synaptic elimination during crucial neurodevelopmental periods has been associated with cortical thinning and cognitive impairments (Hartmann et al., 2024; Toader et al., 2025). Similarly, in depression, peripheral inflammation modifies hypothalamic-pituitary-adrenal (HPA) axis function, resulting in increased glucocorticoid levels and exacerbating immune dysregulation. Chronic inflammation influences metabolic signaling, mitochondrial function, and endothelial integrity, resulting in compromised neurovascular coupling and BBB permeability (Yin et al., 2024). However, neuroinflammatory cascades demonstrate bidirectional interactions with neural circuits, establishing feedback loops that perpetuate maladaptive network states and the chronicity of symptoms. The acknowledgment of immune-mediated influence on neural function has led to the investigation of immunomodulatory therapies, such as anti-cytokine agents, microglial inhibitors, and lifestyle modifications, as supplementary approaches for treatment-resistant depression and psychosis (Huang et al., 2025; Nava et al., 2025). Thus, these findings establish neuroimmune dysregulation as a mechanistic link between peripheral immune activity and central neurotransmission, emphasizing inflammation as a crucial therapeutic target in precision psychiatry.

### 2.5 Mitochondrial and metabolic signaling

Mitochondria are essential for sustaining neuronal function through the integration of energy metabolism, synaptic activity, calcium homeostasis, and redox balance. Recent findings suggest that mitochondrial dysfunction and disrupted metabolic signaling play significant roles in the pathophysiology of psychiatric disorders, such as SCZ, bipolar disorder, MDD, and others (Pinto Payares et al., 2024; Zong et al., 2024). Neurons are energy-dependent cells that primarily utilize oxidative phosphorylation to support action potential generation, neurotransmitter release, and synaptic plasticity. Disruptions in mitochondrial bioenergetics result in inadequate ATP production, modifications in the NAD^+^/NADH ratio, and heightened generation of reactive oxygen species (ROS), all of which undermine neuronal excitability and the fidelity of signaling (Nunes et al., 2025). Additionally, oxidative stress causes additional damage to mitochondrial DNA (mtDNA), lipids, and proteins, leading to a self-reinforcing cycle of dysfunction that worsens synaptic and circuit-level instability. Further, metabolic signaling pathways, including AMP-activated protein kinase (AMPK), mTOR, and peroxisome proliferator-activated receptor gamma coactivator 1-alpha (PGC-1α), concurrently integrate cellular energy status with neuroplastic processes and stress adaptation (Rakshe et al., 2024; Zong et al., 2024). The dysregulation of these pathways negatively affects neuronal resilience and leads to changes in glutamate metabolism, excitotoxicity, and an abnormal balance between excitatory and inhibitory signals. Furthermore, impaired mitochondrial trafficking and fission–fusion dynamics disrupt local energy supply at synaptic terminals, thereby undermining activity-dependent synaptic remodeling (Zaninello et al., 2024). Moreover, mitochondrial dysfunction interacts with neuroinflammatory and neuroimmune processes, thereby amplifying oxidative and metabolic stress. These alterations collectively result in deficits in neuronal excitability, cognitive performance, and mood regulation (Ye et al., 2025). Thus, examining the complex interactions among mitochondrial bioenergetics, oxidative stress, and neural signaling provides essential mechanistic insights into psychiatric pathophysiology and highlights mitochondrial metabolism as a potential therapeutic target for reestablishing cellular and network homeostasis.

## 3 Synaptic and circuit level mechanism

Psychiatric disorders result from disruptions at multiple neural levels, characterized by changes in synaptic structure, abnormal oscillatory signaling, and dysfunctional circuit patterns. Dysregulated synaptic pruning, impaired gamma and theta oscillations, and disrupted connectivity within cortico-striatal, amygdala-prefrontal, and hippocampal networks collectively hinder cognition, emotion, and behavior, highlighting circuit-level targets for precision neuromodulatory and pharmacological interventions (Gong et al., 2024).

### 3.1 Synaptic architecture and connectivity

The integrity of synaptic architecture, both structural and functional, is crucial for effective neuronal communication, cognitive processing, and emotional regulation. Changes in dendritic spine density, synaptogenesis, and synaptic pruning, especially during early neurodevelopmental phases, are critical pathological mechanisms associated with various psychiatric disorders, such as SCZ, ASD, and MDD (Howes et al., 2025). Emerging studies have shown that dendritic spines serve as the main locations for excitatory synaptic transmission and exhibit dynamic remodeling during development and in response to experience-dependent plasticity. Further, disruption of spine formation and elimination regulation undermines the delicate equilibrium between synaptic stability and flexibility essential for adaptive learning and memory (Akhgari et al., 2024). SCZ is characterized by postmortem and imaging studies that consistently demonstrate reduced dendritic spine density in the prefrontal cortex and hippocampus. This reduction is indicative of excessive or mistimed synaptic pruning during adolescence, potentially influenced by overactivation of complement-mediated microglial processes (Howes and Onwordi, 2023). In contrast, ASD is frequently linked to heightened spine density and disrupted synaptic pruning, resulting in excessive excitatory connectivity and suboptimal network integration. Molecular regulators, including BDNF, DISC1, SHANK proteins, and the mTOR signaling pathway, are pivotal in the coordination of synaptogenesis and pruning (Zhang Y. et al., 2024; Kesidou et al., 2025). Their dysregulation leads to structural and functional disorganization of cortical microcircuits. The architectural changes disrupt neuronal synchrony and oscillatory coordination among various brain regions, resulting in impairments in working memory, attention, and social cognition (Soto-Icaza et al., 2024). Additionally, atypical connectivity patterns, namely spanning hyperconnectivity in local circuits to hypoconnectivity in long-range networks were disturb the hierarchical structure of brain communication, which is a characteristic of psychiatric disorders (Xia et al., 2024). Thus, exploring the relationship between molecular and developmental disruptions and their effects on synaptic architecture is essential for elucidating disease mechanisms. This understanding can inform potential therapeutic strategies aimed at synaptic remodeling and restoring connectivity, ultimately enhancing cognitive and emotional outcomes in psychiatric disorders.

### 3.2 Oscillatory and rhythmic signaling

Neuronal oscillations establish the temporal framework that synchronizes communication among brain regions, thereby enhancing perception, cognition, and emotional regulation. Oscillatory dynamics, especially in the gamma (30–100 Hz) and theta (4–8 Hz) frequency bands, play a vital role in synchronizing neural ensembles and facilitating higher-order cognitive and affective processes (Shi et al., 2025). Disruptions in rhythmic patterns have been consistently associated with psychiatric disorders, including SCZ, MDD, and anxiety. In SCZ, diminished gamma-band power and disrupted phase synchronization indicate dysfunction of PV^+^ interneurons and NMDA receptor hypofunction, leading to desynchronized cortical activity and cognitive deficits, such as impairments in working memory and perceptual distortions (Marín, 2024; De Pieri et al., 2025). Further, abnormalities in theta oscillations, crucial for hippocampal-prefrontal communication and memory encoding, lead to deficits in attention, emotional regulation, and episodic memory in MDD and anxiety disorders. Functional neuroimaging and electrophysiological studies indicate that disrupted thalamo-cortical and fronto-limbic synchronization is the basis for these oscillatory abnormalities, resulting in impaired integration among sensory, cognitive, and affective networks (Tsai et al., 2023; Kragel et al., 2025; Tang et al., 2025). In depression, reduced fronto-limbic coherence is associated with emotional dysregulation and rumination, whereas heightened amygdala-prefrontal coupling in anxiety states sustains fear processing and hypervigilance. The thalamus functions as a pacemaker for cortical oscillations and is essential for maintaining rhythmic integrity; its dysregulation leads to global desynchronization and abnormal signal propagation across networks (Huang et al., 2023; Onofrj et al., 2023). Additionally, disruptions in oscillatory signaling indicate underlying synaptic and circuit-level dysfunctions and serve as a mechanistic link between cellular pathology and network-level communication deficits. Emerging neuromodulatory interventions, including transcranial magnetic stimulation (TMS) and deep brain stimulation (DBS), aim to restore oscillatory synchrony (Wang Q. et al., 2024; Dai et al., 2025). Thus, focusing these approaches hold therapeutic potential for reestablishing functional connectivity and enhancing cognitive and affective outcomes in psychiatric disorders.

### 3.3 Neural circuits motifs in psychiatric pathophysiology

The functional organization of the brain is determined by recurrent circuit motifs that integrate sensory input, emotional valence, and cognitive control. The disruption of these motifs, which regulate the flow of information between cortical and subcortical structures, represents a fundamental pathophysiological mechanism associated with psychiatric disorders (Tekin and Cummings, 2002). Additionally, disruption of specific circuit motifs propagates molecular abnormalities to systems-level dysfunction through altered neural oscillations and impaired interregional communication. Cortico–striatal and thalamo–cortical loops rely on precisely timed inhibitory and excitatory signaling to generate gamma- and theta-band oscillations that support cognitive control, working memory, and sensory integration (Buzsáki and Wang, 2012; Lisman and Jensen, 2013). Perturbations in interneuron function or synaptic plasticity reduce oscillatory coherence, leading to desynchronized network activity observed in electrophysiological and neuroimaging studies of psychiatric and neurodegenerative disorders. Importantly, pharmacological or genetic restoration of upstream molecular defects normalizes oscillatory dynamics and circuit coupling, supporting a causal link between molecular dysregulation and network-level impairment rather than simple association (Cho et al., 2006; Gonzalez-Burgos et al., 2015).

The cortico-striatal-thalamo-cortical (CSTC) loops, amygdala-prefrontal circuits, and hippocampal-cortical networks have been consistently associated with the development of distinct but overlapping symptom domains, including compulsivity, mood dysregulation, and cognitive dysfunction (Hudgins et al., 2024; Choe et al., 2025). The CSTC circuitry, responsible for mediating goal-directed behavior, action selection, and inhibitory control, plays a crucial role in obsessive-compulsive disorder (OCD) and SCZ. In OCD, hyperactivity in the orbitofrontal and anterior cingulate cortices, along with dysregulated striatal gating, results in excessive thalamo-cortical feedback, contributing to the emergence of intrusive thoughts and compulsive behaviors (Shephard et al., 2021). In SCZ, altered dopaminergic modulation within the CSTC pathway disrupts the accuracy of reward and salience processing, contributing to psychosis and disorganized cognition. Additionally, the amygdala-prefrontal circuitry, critical for emotional appraisal and regulation, is significantly disrupted in depression and PTSD (Cui et al., 2024; Li Y. et al., 2025). The hypoactivation of the ventromedial prefrontal cortex (vmPFC) and hyperreactivity of the amygdala disrupt top-down inhibitory control, leading to sustained negative affect, increased stress reactivity, and maladaptive fear responses. In PTSD, abnormal connectivity among the amygdala, hippocampus, and medial prefrontal cortex contributes to intrusive memories and deficits in extinction learning (Haris et al., 2023; Davis and Hamner, 2024). Further, the hippocampal-cortical network integrates episodic memory and contextual processing, displaying both structural and functional deficits in various psychiatric disorders. The emerging evidence suggest that reduced hippocampal volume, impaired theta-gamma coupling, and diminished communication with the prefrontal cortex are linked to memory impairment and cognitive inflexibility in depression and SCZ (Černousova and Patrono, 2025). These circuit-level perturbations demonstrate the emergence of psychiatric symptoms from dysfunction within distributed and interconnected neural systems. Thus, comprehending these motifs establishes a mechanistic framework that connects molecular and synaptic abnormalities to observable behavioral phenotypes. It also underscores potential avenues for circuit-targeted therapies, such as neuromodulation, cognitive interventions, and precision pharmacology, intended to restore functional connectivity and network homeostasis.

## 4 Network and systems-level disruption

**FIGURE 3:**
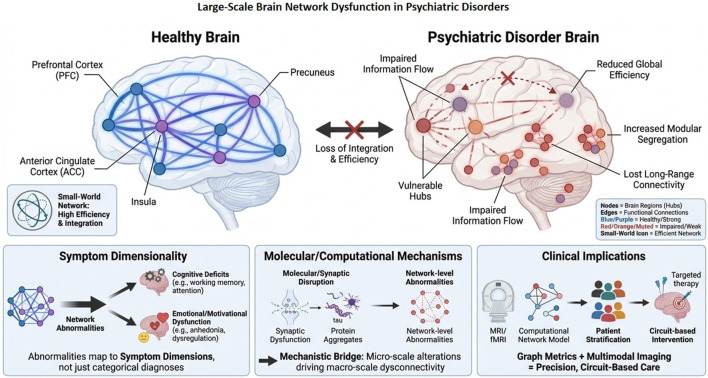
Schematic of large-scale brain network disruption in psychiatric illness. Compared with the efficient small-world organization of the healthy brain, psychiatric illness is characterized by reduced global efficiency, vulnerable hub structure, disrupted information flow, and increased modular separation. Molecular and synaptic disruption scales up to network-level disturbances, which in turn inform symptom dimension and precision, circuit-based intervention strategies.

Psychiatric disorders are characterized by extensive network disruptions rather than isolated abnormalities. Analyses utilizing graph theory and neuroimaging demonstrate topological reorganization, hub vulnerability, and modular segregation that contribute to cognitive and emotional dysfunction. Further, dimensional models based on network connectivity associate changes in connectivity patterns with cognitive, emotional, and motivational processes, providing mechanistic insights and potential predictive biomarkers for precision psychiatry and targeted therapeutic approaches (Xin et al., 2024; Bosl et al., 2025; Mo et al., 2025; Zhang et al., 2025) (Figure 3).

### 4.1 Functional connectivity and graph-theoretic insights

Recent developments in neuroimaging and network neuroscience have shifted the perspective on psychiatric disorders from localized abnormalities to disruptions in large-scale brain connectivity. Functional connectivity, characterized by the temporal correlation of neural activity across various regions, offers a systems-level approach for investigating the disruption of neural communication in mental disorders (Xia et al., 2024). Graph-theoretic modeling has become an effective method for quantifying disruptions by depicting the brain as a complex network of nodes (brain regions) and edges (functional connections). For example, healthy brains demonstrate an organization with small-world properties, which are marked by efficient integration among modules and specialized local processing, thereby optimizing information transfer and cognitive performance (Akın et al., 2024). Psychiatric disorders are characterized by topological reorganization, hub vulnerability, and modular segregation, which together diminish global efficiency and network resilience. Alterations in key network hubs, including the precuneus, anterior cingulate cortex, insula, and dorsolateral prefrontal cortex, have been observed in SCZ, MDD, and bipolar disorder (Chen et al., 2025). These hubs function as integrative relay centers between cognitive and affective systems, exhibiting reduced centrality and weakened connectivity. This results in inefficient information flow and impaired coordination among networks. Increased modular segregation, characterized by diminished inter-network connectivity and improved intra-network coherence, further leads to cognitive rigidity and maladaptive emotional processing (Chan et al., 2024; Wu et al., 2024). In contrast, hyperconnectivity in specific subnetworks, like the default mode network (DMN), may lead to heightened self-referential processing and rumination associated with depression. Additionally, graph-theoretic analyses reveal a disrupted rich-club organization, characterized by preferential connectivity among highly connected hubs, suggesting a deterioration of hierarchical communication crucial for adaptive cognition (Barreiros et al., 2024; Zheng et al., 2024). The identified topological abnormalities are associated with symptom severity, cognitive deficits, and treatment response, highlighting their clinical significance. Further, network topology provides mechanistic insights into the emergent properties of psychiatric dysfunction, where molecular and synaptic disruptions aggregate to modify global brain organization (Choi et al., 2025; Mo et al., 2025). Thus, the integration of graph theory with multimodal imaging and computational modeling establishes a robust framework for the identification of network biomarkers, patient stratification, and the guidance of circuit-level interventions designed to restore connectivity efficiency and network homeostasis in psychiatric disorders.

### 4.2 Network-based predictors of symptoms dimensions

The shift from categorical diagnostic frameworks to dimensional models of psychopathology indicates an increasing acknowledgment that psychiatric symptoms stem from distributed network dysfunctions instead of isolated regional lesions or neurotransmitter irregularities. Network neuroscience offers a comprehensive framework for connecting neural circuitry with behavioral dimensions, including cognition, emotion, and motivation, which are fundamental constructs related to mental health and disease (Pan et al., 2024; Stevanovic et al., 2024; Lett et al., 2025). Alterations in large-scale brain networks demonstrate transdiagnostic patterns that align with symptom dimensions common to multiple disorders, rather than adhering strictly to diagnostic boundaries. Cognitive deficits, prevalent in SCZ, MDD, and bipolar disorder, are linked to impaired connectivity within and between the frontoparietal control network and the dorsolateral prefrontal cortex, which are critical for executive functioning, working memory, and cognitive flexibility (Yang J. et al., 2024). Abnormalities in emotion-related networks, such as the amygdala-prefrontal and salience networks, contribute to affective instability, hypervigilance, and impaired emotional regulation seen in anxiety and mood disorders. Dysregulated interactions between the DMN and salience network contribute to maladaptive self-referential processing and rumination in depression (Willinger et al., 2024; Langhammer et al., 2025). Additionally, aberrant fronto-striatal connectivity disrupts reward valuation and motivation in anhedonia and apathy across various disorders. The findings endorse a continuum-based framework wherein the severity and combination of symptoms indicate graded changes in network topology, connectivity strength, and dynamic flexibility, rather than categorical distinctions (Derosiere et al., 2025). Advanced computational and connectomic methods, including graph theory, dynamic causal modeling, and machine learning (ML), facilitate the identification of individualized network signatures that predict specific symptom dimensions and treatment responsiveness. Further, functional connectivity patterns within the prefrontal-limbic circuitry have been demonstrated to predict outcomes of antidepressant and cognitive behavioral therapy, whereas fronto-striatal dynamics are indicative of motivational enhancement following dopaminergic modulation (Jiao et al., 2025; Liu et al., 2025). These insights contribute to a precision psychiatry framework in which network-level biomarkers act as objective indicators of symptom expression, disease progression, and treatment response. Dimensional models informed by network neuroscience provide a mechanistic and clinically relevant understanding of psychiatric disorders by connecting cognition, emotion, and motivation to dynamic network interactions.

### 4.3 Network-based predictors of symptom dimensions: shared and disorder-specific features

While SCZ, MDD, and bipolar disorder share similar abnormalities in networks at a network level (e.g., prefrontal-limbic, salience, and default) there is also considerable heterogeneity between these disorders regarding their developmental trajectories, regions of vulnerability, and molecular mechanisms. For instance, SCZ is considered to be a neurodevelopmental disorder, with genetic risk factors present during childhood as well as immune insults occurring prenatally, in addition to excessive synaptic pruning occurring during early childhood, producing significant disruptions to the thalamo-cortical and fronto-striatal networks. Each of these network disruptions is associated with (1) decreased functioning of NMDA receptors, (2) altered signaling of parvalbumin-positive interneurons, and (3) an imbalance between excitation and inhibition in these networks; collectively, these disruptions lead to deficits in cognitive control and working memory (Homayoun and Moghaddam, 2007; Rapoport et al., 2012). Moreover, compared to other forms of depressive disorders, MDD has a much less homogeneous pattern of presentation and many times is related to periods of stress throughout life, from adolescence or into adulthood. The MDD phenotype(s) are characterized by hyper-connectivity within the default mode network and decreased functional connectivity between prefrontal regulatory circuits and limbic structures (for example, the amygdala and hippocampus). These circuit-level abnormalities develop from the alteration of monoaminergic signaling, neurotrophic support (for example BDNF), mutations in epigenetic regulation and low-grade chronic inflammation, which may lead to dysregulation of emotion(s) and result in poor emotional resilience (Castrén and Rantamäki, 2010; Nestler, 2014; Kaiser et al., 2015). In contrast, bipolar disorder is somewhere in between, as it presents both genetically or developmentally vulnerable individuals who have episodes of large periods of network instability across their lives (both unipolar circuitry and bipolar). Studies involving functional imaging have shown state-dependent differences in the prefrontal-limbic or fronto-striatal circuits of individuals with bipolar disorder, with patterns of connectivity and oscillatory dynamic differences depending upon whether the person is at a manic or depressive phase. At the molecular level, bipolar disorder is associated with alterations in intracellular signalling pathway regulation (for example, GSK3β and calcium signalling), dysfunction in the mitochondria, and alterations in circadian rhythms (Strakowski et al., 2005; Beaulieu et al., 2008; McClung, 2013; Insel, 2014). These three areas of dysregulation help to create the oscillatory and state-dependent nature of the network dysregulation patterns seen in bipolar disorder (Table 2). Based on these observations, we suggest that (i) shared network motifs offer a transdiagnostic basis for symptom dimensions; (ii) however, the timing of development, susceptibility of regional circuits to disease, and molecular pathways associated with a disorder all play an important role in shaping clinical phenotype. Therefore, when developing a model of potential treatments using network-based approaches, it is necessary to consider the unique features of an individual disorder to inform intervention strategies that are tailored to the specific disorder.

**TABLE 2:** Network-based similarities and disorder-specific heterogeneity across psychiatric disorders.

Feature	Schizophrenia (SCZ)	Major depressive disorder (MDD)	Bipolar disorder
Primary developmental trajectory	Neurodevelopmental; early-life genetic and prenatal immune risk	Stress-related; adolescent or adult onset	Mixed; developmental vulnerability with episodic course
Preferentially affected brain regions	Prefrontal cortex, thalamus, hippocampus, striatum	Prefrontal cortex, amygdala, hippocampus, default mode network	Prefrontal–limbic and fronto-striatal circuits
Dominant network dysfunction	Thalamo–cortical and fronto-striatal dysconnectivity; reduced gamma synchrony	DMN hyperconnectivity; reduced prefrontal–limbic coupling	State-dependent network instability (mania vs. depression)
Key molecular drivers	NMDA receptor hypofunction; PV interneuron dysfunction; synaptic pruning abnormalities	Monoaminergic dysregulation; reduced BDNF; epigenetic and inflammatory signaling	GSK3β and calcium signaling dysregulation; mitochondrial and circadian abnormalities
Oscillatory alterations	Impaired gamma and theta oscillations	Altered low-frequency oscillations; reduced network flexibility	Mood-state–dependent oscillatory changes
Core symptom dimensions linked to networks	Cognitive control and working memory deficits	Affective dysregulation and rumination	Mood instability and impaired emotional regulation
Transdiagnostic overlap	E/I imbalance; prefrontal network disruption	Prefrontal–limbic dysregulation	Shared prefrontal–limbic circuit vulnerability

## 5 Genetic, epigenetic, and developmental regulation of neural signaling

**FIGURE 4:**
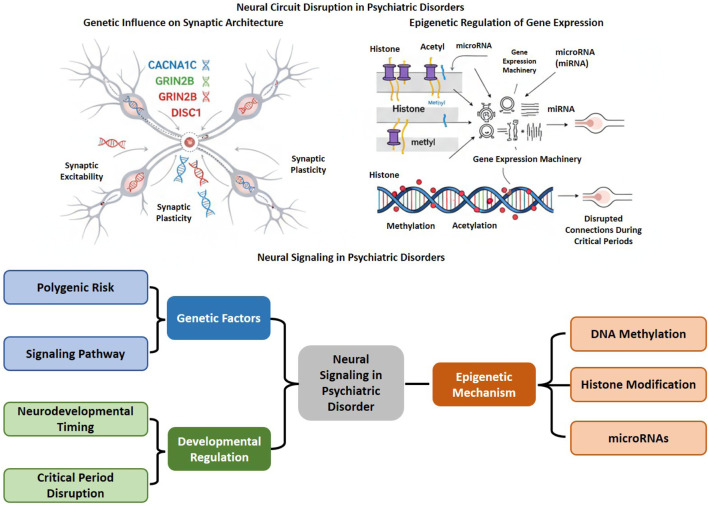
Genetic and epigenetic modulation of neural circuit impairment in mental disorders: Genetic variations (e.g., *CACNA1C*, *GRIN2B*, *DISC1*) affect synaptic excitability and plasticity, leading to modified neuronal signaling and impaired synaptic structure. Epigenetic processes, including DNA methylation, histone modification, and microRNA-mediated control, influence gene expression and connection throughout essential neurodevelopmental phases. The interplay of polygenic risk, signaling pathways, and developmental timing contributes to brain circuit dysregulation, underscoring the convergence of genetic and epigenetic variables in the pathophysiology of psychiatric diseases.

Neural signaling in psychiatric disorders is intricately regulated by genetic, epigenetic, and developmental factors. Polygenic variants in synaptic genes, such as *CACNA1C*, *GRIN2B*, and *DISC1*, affect excitability and plasticity. Epigenetic mechanisms, including DNA methylation, histone modification, and microRNAs (miRNA), dynamically influence gene expression and synaptic function (Benatti et al., 2024; Peña, 2026). Disruptions in signaling during critical periods of neurodevelopment can alter circuit maturation, leading to long-term vulnerabilities that contribute to cognitive, emotional, and behavioral dysfunction in psychiatric conditions (Figure 4).

### 5.1 Polygenic risk and signaling pathway enrichment

Psychiatric disorders exhibit a high degree of polygenicity, where risk is influenced by the cumulative impact of multiple common genetic variants, each contributing a small effect size that collectively modifies neural signaling, synaptic plasticity, and circuit organization (Ayano et al., 2025). Genome-wide association studies (GWAS) have identified numerous susceptibility loci across disorders including SCZ, bipolar disorder, MDD, and ASD. These studies demonstrate significant enrichment in genes related to synaptic structure, neurotransmission, and intracellular signaling pathways. Key genes, including *CACNA1C*, *GRIN2B*, and *DISC1*, are crucial in the regulation of neuronal excitability, calcium signaling, and synaptic plasticity (Astorino et al., 2025). For instance, variants in *CACNA1C*, encoding the α1C subunit of L-type voltage-gated calcium channels, affect intracellular calcium dynamics essential for neurotransmitter release and activity-dependent gene expression via CREB and MAPK/ERK signaling pathways. The dysregulation of *CACNA1C* expression or channel kinetics is associated with impaired synaptic integration, abnormal emotional processing, and disrupted prefrontal-limbic connectivity in bipolar disorder and SCZ (Datta et al., 2024; Boller et al., 2025). Further, polymorphisms in *GRIN2B*, which encodes the NR2B subunit of the NMDA receptor, influence glutamatergic transmission, LTP, and the excitatory-inhibitory balance, thereby contributing to cognitive and perceptual disturbances associated with psychotic and developmental disorders (Tumdam et al., 2024). The *DISC1* gene is implicated in various neuropsychiatric disorders, including SCZ. Its role in neuronal development and synaptic function has garnered significant research interest. The gene, initially identified in a Scottish family exhibiting a high prevalence of psychiatric disorders, regulates neurodevelopmental processes, including neuronal migration, axonal growth, and synapse formation, via its interaction with signaling pathways, such as cAMP-PKA and PI3K-Akt-mTOR (Gutiérrez-Rodríguez et al., 2025; Volgin et al., 2025). Functional variants and structural rearrangements in *DISC1* disrupt mitochondrial trafficking, cytoskeletal organization, and synaptic connectivity, resulting in modified cortical circuitry and cognitive impairment. Pathway-level analyses indicate that polygenic risk converges on signaling networks related to calcium homeostasis, glutamate receptor trafficking, and neurotrophic regulation, specifically the BDNF-TrkB and Wnt pathways (Norkett et al., 2020; Schirò et al., 2022). The widespread effects of these variants highlight that psychiatric vulnerability arises not from isolated gene mutations but from disruptions at the network level within interconnected molecular pathways that regulate synaptic signaling and neurodevelopment. The integration of polygenic risk scores with transcriptomic and functional imaging data facilitates the mechanistic mapping of gene networks to brain connectivity, providing a biologically informed framework for precision psychiatry and genetically guided therapeutic interventions (Wang M. et al., 2024; Bracher-Smith and Escott-Price, 2025).

### 5.2 Epigenetic mechanism

Epigenetic regulation serves as a vital link among genetic predisposition, environmental influences, and neural function, facilitating the dynamic adjustment of gene expression without modifying the DNA sequence itself. Evidence suggests that DNA methylation, histone modifications, and miRNA-mediated post-transcriptional regulation are significant mechanisms involved in the dysregulation of neural signaling, synaptic plasticity, and behavioral abnormalities in psychiatric disorders (Ohi et al., 2025; Peña, 2026). For instance, DNA methylation, which generally takes place at cytosine-phosphate-guanine (CpG) dinucleotides, influences chromatin accessibility and gene transcription. Aberrant methylation patterns in synaptic and neurodevelopmental genes, including *BDNF*, *RELN*, *GAD1*, and *GRIN2B*, have been observed in SCZ, MDD, and bipolar disorder, frequently resulting in transcriptional repression of genes critical for synaptic integrity and neuronal communication (Saada and Stern, 2025; Yu et al., 2025). Additionally, environmental stressors, such as early-life adversity and chronic stress, can lead to lasting hypermethylation of glucocorticoid receptor and neurotrophic factor promoters, consequently impairing the regulation of the HPA axis and synaptic resilience (Rizavi et al., 2023). Histone modifications, especially acetylation and methylation, impact chromatin structure and transcriptional dynamics by modifying DNA accessibility to transcription factors. Reduced histone acetylation at the promoters of plasticity-related genes correlates with diminished expression of synaptic proteins and cognitive impairment (Mai et al., 2025). In contrast, inhibition of histone deacetylases (HDACs) has been demonstrated to counteract these effects, leading to the restoration of synaptic strength and behavioral flexibility in preclinical models (Kilgore et al., 2010; Gräff et al., 2012). In addition to these mechanisms, miRNAs regulate gene expression by targeting messenger RNAs for degradation or translational repression (Garayo-Larrea et al., 2024). For example, dysregulated miRNAs, including miR-132, miR-137, and miR-124, are associated with neuronal differentiation, synaptogenesis, and the expression of neurotransmitter receptors. miR-137, identified as a risk locus for SCZ, modulates genes related to calcium signaling and vesicular transport, thereby connecting molecular regulation to deficits in network-level communication (Pergola et al., 2024; Adly et al., 2025). These epigenetic modifications collectively integrate environmental and developmental signals, influencing neural circuitry and behavior. Their reversible nature presents significant therapeutic potential; pharmacological interventions aimed at epigenetic enzymes or miRNA pathways may correct abnormal gene expression patterns and synaptic function, facilitating the advancement of mechanism-based treatments in precision psychiatry.

### 5.3 Neurodevelopment timing and critical period disruption

Neurodevelopment is regulated by meticulously timed molecular and cellular mechanisms that coordinate neuronal differentiation, synaptogenesis, and circuit refinement. Perturbations during critical periods of brain maturation, characterized by increased plasticity and sensitivity to environmental input, can have significant and lasting impacts on brain structure and function, increasing the risk of psychiatric disorders (Khodosevich and Sellgren, 2023; Vivi and Di Benedetto, 2024). Disruptions in early-life signaling, such as abnormal neurotransmitter dynamics, neurotrophic imbalances, inflammatory activation, and hormonal dysregulation, can impede the sequential development of excitatory and inhibitory circuits critical for cognitive, emotional, and social processing (Caballero et al., 2021). Further, abnormal glutamatergic and GABAergic signaling during perinatal and adolescent stages disrupts the establishment of excitatory-inhibitory balance, which impairs cortical maturation and prefrontal-limbic connectivity, which are key features of SCZ, ASD, and mood disorders (Hollestein et al., 2023). Additionally, altered BDNF-TrkB signaling during synaptic refinement phases can disrupt dendritic spine stability and synaptic pruning, resulting in hyperconnectivity or inefficient circuit integration seen in neurodevelopmental psychopathologies (Hernández-del Caño et al., 2024). Early-life stress, infection, or nutritional deficiencies induce inflammatory and endocrine responses that alter neurodevelopmental trajectories through epigenetic programming and microglial activation, resulting in enduring impacts on neuronal excitability and synaptic organization (Rahman and McGowan, 2022). Disruption during critical periods affects the development of extensive networks, including the default mode and salience networks, which are essential for advanced cognitive functions and emotional regulation. The timing of perturbation is crucial in determining vulnerability; disruptions during early gestation can impair neurogenesis and migration, while adolescent disturbances typically influence myelination, synaptic pruning, and the maturation of neurotransmitter receptors (Azarias et al., 2025; Banihashemi et al., 2025). Longitudinal imaging and transcriptomic studies indicate that delayed or excessive pruning, atypical myelination, and impaired synaptic stabilization are factors contributing to network inefficiency and cognitive decline in psychiatric disorders (Hansen et al., 2022). The findings indicate that mental disorders, while frequently clinically evident during adolescence or early adulthood, may stem from subtle variations in developmental timing that predispose individuals to later dysfunction. Thus, exploring the impact of early-life signaling abnormalities on persistent circuit vulnerabilities provides essential insights for developing preventive strategies and early interventions that aim to restore neurodevelopmental trajectories and improve neural resilience in at-risk populations.

## 6 Computation and artificial intelligence-driven insights into neural signaling

**FIGURE 5:**
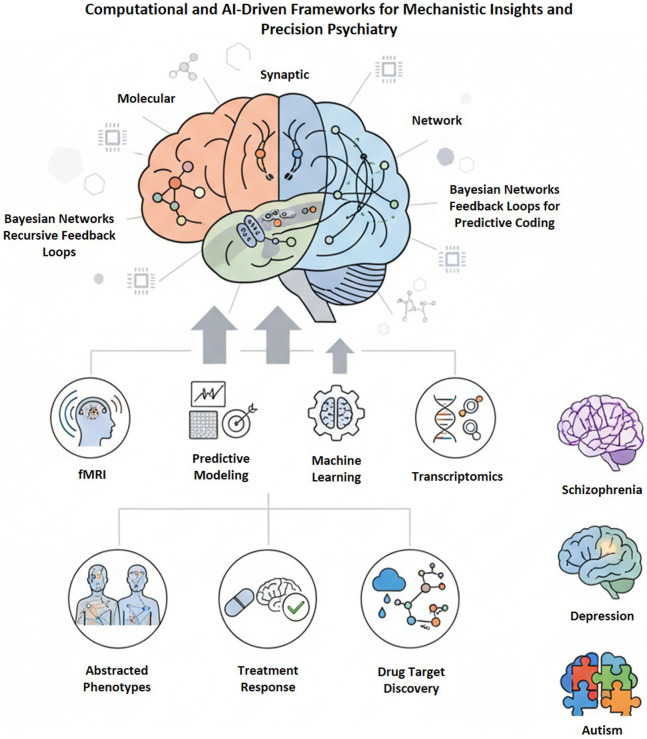
Computational and AI-based frameworks for mechanistic understanding and precision psychiatry. The integration of molecular, synaptic, and network-level data using Bayesian networks and recursive feedback loops facilitates predictive modeling of brain activity. Multimodal inputs, including fMRI, machine learning, and transcriptomics, contribute to dynamic models of mental diseases, such as SCZ, depression, and autism. These computer methods enable the abstraction of phenotypes, the prediction of treatment responses, and the identification of pharmacological targets. Additionally, AI-based models enhance individualized therapies and data-driven methods in precision psychiatry by correlating mechanistic brain processes with clinical results.

Computational and AI-driven methodologies are transforming psychiatry by connecting molecular, synaptic, and network dysfunctions via predictive modeling, multimodal data integration, and ML techniques. Bayesian and predictive coding models characterize psychiatric symptoms as inference errors, whereas multimodal connectomics incorporates fMRI, EEG, PET, and transcriptomics (Monosov et al., 2024; Huang and Shu, 2025; Sun J. et al., 2025). AI facilitates mechanistic discovery, network-based phenotyping, and precision drug targeting, thereby enhancing personalized neurotherapeutics (Figure 5).

### 6.1 Computational psychiatry models of signal integration

Computational psychiatry provides a mechanistic framework for understanding the relationship between neural signaling disruptions and the emergence of aberrant cognition and behavior through the application of quantitative models of brain function. This approach is centered on Bayesian brain models, predictive coding, and hierarchical inference frameworks, which view the brain as a probabilistic inference machine that consistently updates internal models to predict sensory inputs and reduce uncertainty (Qela et al., 2025). Perception, cognition, and emotion arise from the interplay between bottom-up sensory information and top-down prior expectations. Psychiatric disorders can be conceptualized as disorders of inference and prediction, wherein abnormalities in neural signaling affect the precision weighting of sensory information relative to prior knowledge (Keller and Sterzer, 2024). In SCZ, impaired NMDA receptor function and cortical disinhibition may result in the aberrant assignment of salience to irrelevant stimuli, leading to delusions and hallucinations via disrupted predictive coding. In depression, maladaptive priors that reflect negative expectations skew hierarchical inference, leading to enduring pessimism and a failure to revise beliefs in light of positive evidence (Keller and Sterzer, 2024; Bottemanne et al., 2025). In anxiety, heightened precision of threat-related priors increases prediction errors in the amygdala-prefrontal circuitry, thereby maintaining hypervigilance and anticipatory fear. Hierarchical Bayesian inference-based computational models correlate dysfunctions with quantifiable neural dynamics, including changes in cortical oscillations, synaptic gain modulation, and network connectivity, thereby integrating molecular, circuit-level, and behavioral data (Hess et al., 2025). Moreover, in depression, computational models describe maladaptive priors and impaired belief updating that bias hierarchical inference toward pessimistic expectations and reduced reward sensitivity, while in anxiety disorders excessive precision of threat-related priors amplifies prediction errors within amygdala–prefrontal circuits, sustaining hypervigilance and avoidance behaviors (Browning et al., 2015; Huys et al., 2015; Kube et al., 2020). These models offer a cohesive framework for the integration of neuroimaging, electrophysiology, and behavioral data, facilitating the individualized characterization of computational phenotypes that surpass conventional diagnostic classifications. Furthermore, computational frameworks possess significant clinical implications; by identifying latent parameters that influence perception, learning, and decision-making, they can forecast treatment responses and inform personalized interventions (Karvelis et al., 2023). Bayesian and predictive coding models reconceptualize psychiatric disorders as disruptions in the brain’s inferential architecture, connecting abnormal neural signaling to distorted mental representations and facilitating the advancement of precision computational psychiatry. Empirical support for this framework comes from computational studies demonstrating that aberrant precision weighting within hierarchical predictive coding models can reproduce key psychotic phenomena, including hallucinations and delusions, when NMDA receptor–dependent synaptic gain is disrupted (Sterzer et al., 2018).

### 6.2 Multimodal data fusion and connectomic modeling

Multimodal data fusion and connectomic modeling are advanced methodologies in neuroscience that facilitate a thorough comprehension of neural signaling hierarchies at molecular, cellular, and systems levels. Integrating complementary modalities, including functional magnetic resonance imaging (fMRI), electroencephalography (EEG), positron emission tomography (PET), and single-cell transcriptomics, enables researchers to develop multiscale models that connect molecular activity with dynamic brain networks and behavioral outcomes (Sui et al., 2023; Ghosh et al., 2024). fMRI delivers high spatial resolution for the identification of distributed functional networks, whereas EEG provides millisecond-level temporal precision to detect oscillatory synchronization and transient communication among neural ensembles. Likewise, PET imaging provides quantitative insights into the distribution of neurotransmitter receptors, metabolic activity, and neuroinflammatory processes, thereby connecting molecular signaling with functional dynamics (Lawn et al., 2023; Tuan et al., 2025). Single-cell transcriptomic data reveal cellular heterogeneity and gene expression patterns that underlie network organization, providing a molecular basis for the observed differences at the circuit level. Integrative computational frameworks, such as graph-theoretic modeling, dynamic causal modeling, and ML-based multimodal fusion, are increasingly employed to synthesize heterogeneous data types into coherent representations of neural function (Piwecka et al., 2023; Yao et al., 2025). These models clarify the mechanisms by which disturbances in synaptic signaling or neurotransmitter systems influence mesoscale circuits, leading to significant network changes associated with psychiatric disorders. The integration of fMRI-derived connectivity with PET measures of dopaminergic or glutamatergic activity facilitates the identification of neurochemical correlates associated with aberrant network synchronization in SCZ and depression (Millevert et al., 2023; Varvari et al., 2025). The integration of EEG oscillatory patterns with single-cell transcriptomic signatures elucidates the translation of cellular-level channelopathies or receptor dysregulation into macroscopic network instability. In addition to providing mechanistic insights, multimodal connectomic models possess diagnostic and therapeutic potential by offering individualized network biomarkers that can predict treatment response or disease progression (Jimenez-Marin et al., 2024; Krejcar and Namazi, 2025). In addition, representative multimodal studies have demonstrated the power of this approach, showing that PET-derived neurotransmitter receptor distributions constrain fMRI network organization and EEG oscillatory synchronization, while integration with transcriptomic data reveals how molecular and cellular perturbations propagate hierarchically to circuit- and systems-level dysfunction (Babiloni et al., 2004; Riedl et al., 2014). Advancements in the field indicate that multimodal data fusion is shifting psychiatry from a symptom-focused approach to one centered on circuits and mechanisms. This transition facilitates the mapping of brain-wide signaling hierarchies that support cognition, emotion, and behavior in both health and disease contexts.

### 6.3 Machine learning in mechanistic discovery and drug targeting

The integration of ML and AI into neuroscience and psychiatry has transformed mechanistic discovery and therapeutic innovation by connecting molecular perturbations with systems-level network dysfunction. AI-driven models analyze complex, high-dimensional datasets from multimodal neuroimaging, genomics, electrophysiology, and clinical phenotyping to reveal latent relationships that traditional analytical methods may overlook (Baydili et al., 2025; Bracher-Smith and Escott-Price, 2025). In psychiatric disorders, the heterogeneity and overlapping symptom dimensions complicate diagnosis and treatment. Further, ML facilitates the identification of network phenotypes, which are distinct patterns of connectivity, activity, and molecular signaling that characterize specific pathophysiological subtypes (Chen et al., 2022). Supervised and unsupervised learning algorithms, including convolutional neural networks (CNNs), graph neural networks (GNNs), and manifold learning approaches, are increasingly employed to correlate functional and structural connectivity abnormalities with underlying molecular signatures obtained from transcriptomic, proteomic, and metabolomic data (Tanaka, 2025). These models clarify the role of disruptions in specific neurotransmitter systems, ion channels, or intracellular signaling pathways (such as mTOR, PI3K-Akt, or MAPK/ERK) in the large-scale network disintegration associated with SCZ, MDD, and ASD (Angela Asir et al., 2025). Additionally, AI enhances mechanistic drug discovery through the integration of pharmacogenomic data with network-level representations, enabling the prediction of therapeutic targets and drug-response profiles. Network-based ML models can identify compounds that rectify abnormal connectivity or signaling patterns by simulating their effects across molecular and circuit hierarchies, a methodology referred to as network pharmacology (Zhang P. et al., 2024; Cui et al., 2025). Moreover, traditional diagnostic categories are less effective for classifying patients when using supervised/unsupervised ML methods, including random forests, support vector machines and deep neural networks, to integrate and use neuroimaging, transcriptomic, epigenomic and electrophysiological data. For instance, multimodal classifiers developed by combining functional connectivity with gene expression profiles have produced the ability to distinguish biologically distinct forms of depression and SCZ having different clinical characteristics, such as site of treatment and rate of response, by gathering data from multiple modalities together (Richiardi et al., 2015; Clementz et al., 2016). Similarly, ML based methods have been developed using resting-state fMRI and EEG characteristics, to be able to predict response to antidepressant and antipsychotic medications, highlighting potential for translational research regarding network-defined biomarkers. Additionally, the importance of using a multi-modal approach allows patterns to be identified that provide converging evidence of dysfunction from a variety of molecular, cellular, and multi-cellular levels (Chekroud et al., 2016; Marquand et al., 2016; Kang and Cho, 2020). In this manner signaling fingerprints are a means of identifying a coordinated level of dysregulation across all of these modalities, thus allowing for the identification of upstream drivers, such as neuroinflammatory processes associated with MDD or SCZ, and downstream phenotypes related to neuronal or synaptic dysfunction. Additionally, these fingerprints provide an avenue of stratifying patients based on biological factors that are causally associated with their treatment responses (Woo et al., 2017).

Emerging studies have demonstrated that deep learning frameworks utilizing extensive pharmacological datasets are capable of predicting ligand-receptor interactions, off-target effects, and opportunities for drug repurposing in neuropsychiatric conditions. The integration of functional MRI connectivity fingerprints with transcriptomic atlases has facilitated the identification of compounds that influence specific neurotransmitter pathways associated with dysfunctional brain networks (Lv et al., 2024; Gupta et al., 2025). Further, reinforcement learning and causal modeling approaches are employed to create adaptive treatment strategies that adjust interventions dynamically based on real-time neural feedback. Moreover, AI-driven mechanistic modeling enhances precision in target identification and contributes to biomarker discovery, facilitating personalized treatment based on an individual’s molecular and network profile (Wright and Anticevic, 2024). With the ongoing increase in computational power and multimodal datasets, ML emerges as a transformative element in psychiatry. It provides a data-driven framework for deciphering neural complexity, enhancing drug discovery, and initiating a new phase of precision neurotherapeutics based on mechanistic insights. The current limitations of AI for clinical purposes represent areas where improvement can be achieved. Most of the current research in this area has focused on smaller to moderate sized cohort studies which raise issues of model overfitting and generalization. The variability in data acquisition protocols, preprocessing pipeline design and feature selection across study sites limits reproducibility among study sites (Marek et al., 2022; Varoquaux and Cheplygina, 2022). Most of the current AI models have a “black box” nature causing challenges with respect to the biological interpretability of the data as well as issues with gaining regulatory approval. To overcome these issues and create effective clinical applications of AI, larger and harmonized multi-site cohort studies must occur. Additionally, standardized analytic methods and continued focus on the use of explainable AI will be needed to maintain the biological basis for any findings and create clinically useful solutions (Topol, 2019; Poldrack et al., 2020). Creating longitudinal multi-omics datasets and linking the resulting data to clinical outcomes will be critical to validating the clinical application of the resulting AI models as they will provide a means of establishing causation or predicting disease progression.

## 7 Translational implications and therapeutic horizons

**FIGURE 6:**
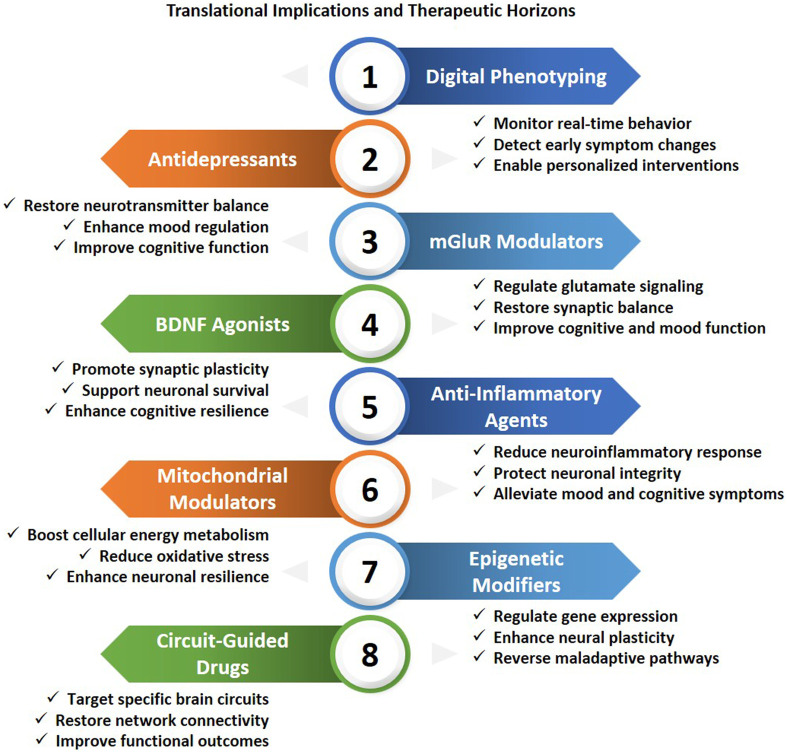
Translational implications and therapeutic prospects in neuropsychiatric diseases: Innovative treatments encompass targets from molecular to systems levels, combining digital and pharmaceutical approaches. Digital phenotyping facilitates immediate behavioral observation and tailored responses. Pharmacotherapies, including antidepressants, mGluR modulators, BDNF agonists, anti-inflammatory medicines, and mitochondrial agents, restore neurotransmission, augment synaptic plasticity, and diminish neuroinflammation. Epigenetic modifiers govern gene expression and neuronal plasticity, whereas circuit-targeted pharmaceuticals seek to reestablish network connection and functional results. These techniques collectively signify an advancing foundation for precision and mechanism-based therapies in psychiatry.

The integration of network neuroscience, molecular psychiatry, and computational modeling is revolutionizing psychiatric care. Translational research currently connects molecular signaling abnormalities to large-scale circuit dysfunctions, thereby advancing mechanism-driven, precision-based treatments. Emerging strategies, such as neuromodulation, molecular therapeutics, circuit-guided pharmacology, and digital biomarkers seek to restore neural communication, maintain network integrity, and achieve the physiological balance necessary for cognition, emotion, and behavior (Komatsu et al., 2021; Tsikonofilos et al., 2025) (Figure 6; Table 3). At the behavioral level, large-scale network instability manifests as deficits in cognitive flexibility, emotional regulation, and adaptive behavior. Functional imaging studies consistently demonstrate that disrupted connectivity within prefrontal–limbic and default mode networks correlate with symptom severity. Crucially, interventions that target upstream molecular mechanisms, such as HDAC inhibition, anti-inflammatory modulation, or restoration of neurotransmitter balance, which can rescue circuit synchronization and improve behavioral outcomes in preclinical models (Grace, 2016; Deco et al., 2017). These findings support a mechanistic cascade in which molecular and cellular perturbations initiate circuit-level dysfunction that scales to network disorganization and clinical phenotypes. Together, this cross-scale framework provides a causal, rather than purely descriptive, account of how molecular pathology translates into systems-level brain dysfunction (Insel, 2014).

**TABLE 3:** Mapping of therapeutic strategies to molecular and network-level targets in neuropsychiatric disorders.

Therapeutic strategy	Molecular targets	Network/Circuit targets	Mechanistic rationale (pathway linkage)	Experimental/Clinical evidence	Translational advantage	Key limitations/Challenges
Repetitive transcranial magnetic stimulation (rTMS)	Modulates glutamatergic and GABAergic neurotransmission; increases BDNF-TrkB signaling	DLPFC, fronto-limbic and salience networks	Restores excitatory–inhibitory balance and network synchrony; normalizes prefrontal–amygdala coupling	RCTs in MDD, SCZ, OCD show improved mood and cognition	Noninvasive network-specific modulation	Response heterogeneity; optimization of stimulation site/frequency
Transcranial direct current stimulation (tDCS)	Alters NMDA receptor activity, voltage-gated channels, and intracellular cAMP-PKA signaling	Prefrontal–parietal executive control network	Enhances plasticity, facilitates synaptic potentiation, strengthens top-down control	Pilot trials show cognitive and affective benefits	Portable, safe adjunct to therapy	Modest efficacy; dose and montage variability
Deep brain stimulation (DBS, adaptive/closed-loop)	Modulates dopamine and serotonin release; affects PI3K-Akt and CREB signaling	Subgenual cingulate, nucleus accumbens, CSTC loops	Direct modulation of pathological oscillations and reward circuits	Clinical benefit in TRD, OCD; adaptive DBS in trials	Circuit-specific, reversible modulation	Invasive; individual variability in network anatomy
Ketamine/NMDA receptor modulators	NMDA receptor antagonism; downstream mTOR-BDNF activation	Normalizes default mode and limbic connectivity; enhances gamma synchrony	Rapid synaptogenesis and restoration of excitatory–inhibitory equilibrium	Rapid antidepressant effect in TRD	Restores plasticity rapidly; mechanistic clarity	Short duration; dissociative effects; abuse potential
mGluR2/3 and mGluR5 modulators	mGluR autoreceptors; modulate glutamate release and PI3K-Akt cascades	Cortico-thalamic and prefrontal networks	Dampens hyperglutamatergic signaling and improves information gating	Preclinical SCZ and anxiety models	Fine-tuned glutamate modulation	Inconsistent clinical efficacy; narrow window
Monoaminergic antidepressants and antipsychotics	SERT, DAT, D2, 5-HT1A, 5-HT2A; downstream cAMP-CREB	Fronto-limbic, salience, and reward networks	Restores neuromodulatory tone; rebalances fronto-limbic and DMN circuits	Extensive clinical efficacy data	Established mechanisms and safety	Delayed onset; incomplete symptom control
BDNF/TrkB agonists and neurotrophic enhancers	BDNF, TrkB, MAPK/ERK, Akt-mTOR	Hippocampal–prefrontal and cognitive control networks	Enhances synaptic growth and dendritic spine density; strengthens connectivity	Preclinical antidepressant and neuroplasticity studies	Directly targets plasticity deficits	BBB penetration issues; clinical validation needed
Anti-inflammatory/immunomodulatory agents	IL-6, TNF-α, microglial signaling, complement (C4A)	DMN and fronto-limbic networks affected by inflammation	Reduces neuroinflammatory-driven pruning and oxidative stress; stabilizes connectivity	Minocycline, cytokine inhibitors in MDD/SCZ adjunct trials	Targets inflammation-linked pathophysiology	Variable immune profiles; systemic risks
Mitochondrial and metabolic modulators	AMPK, PGC-1α, NAD^+^, mTOR	Energy-deficient limbic and executive networks	Restores ATP production, reduces ROS; improves oscillatory stability	Pilot studies in bipolar disorder and depression	Addresses core bioenergetic dysfunction	Limited human validation; metabolic side effects
Ion channel modulators (Ca^2+^, K^+^, Na^+^)	CACNA1C, KCNH2, KCNN3	Cortical microcircuits; oscillatory and thalamo-cortical loops	Normalizes firing precision and rhythmic synchrony	Genetic and electrophysiological evidence in SCZ, bipolar	Mechanistic precision (channelopathies)	Cardiac side effects; narrow therapeutic index
Epigenetic modifiers (HDAC inhibitors, miRNA therapy)	HDAC1/2, DNMT1, miR-137, miR-132	Broad network restoration via transcriptional plasticity	Reactivates silenced neuroplasticity genes; restores excitatory–inhibitory balance	Preclinical behavioral recovery models	Durable molecular resetting potential	Off-target effects; delivery to brain challenging
Circuit-guided drug development	Drug-receptor interaction mapping via connectomic biomarkers	Circuit-specific pharmacological modulation (DMN, CEN, SN)	Aligns receptor action with network topology for targeted restoration	PET-fMRI based precision pharmacology studies	Mechanistic personalization; cross-scale validation	Requires high-quality multimodal datasets
Artificial intelligence-guided adaptive neuromodulation	Data-driven target mapping (BDNF, NMDA, PI3K-Akt)	Closed-loop network reconfiguration based on real-time feedback	ML identifies optimal stimulation patterns to restore network homeostasis	Emerging preclinical and clinical pilot trials	Dynamic, personalized, self-correcting systems	Requires continuous monitoring and safety validation
Behavioral and digital phenotyping-based therapy	Indirectly modulates stress, HPA-axis, and BDNF pathways	Strengthens fronto-parietal, limbic, and salience networks	Experience-dependent plasticity reinforced via digital feedback loops	CBT and digital tracking trials	Non-invasive, scalable, precision monitoring	Adherence and data privacy concerns

### 7.1 Precision neuromodulation

Recent advances in precision neuromodulation technologies, including TMS, transcranial direct current stimulation (tDCS), DBS, and emerging closed-loop systems were representing a major step forward in the ability to selectively target and modulate dysfunctional brain circuits with increasing spatial, temporal, and mechanistic specificity (Stagg and Nitsche, 2011; Fox et al., 2012; Lozano et al., 2019; Soleimani et al., 2023). These approaches are grounded in a network-based understanding of psychiatric and neurological disorders, which conceptualizes disease states as arising from maladaptive interactions across distributed neural systems rather than focal lesions. By directly engaging circuit dynamics, modern neuromodulatory strategies offer a powerful means to restore aberrant communication patterns underlying cognitive, affective, and behavioral dysfunction (Bullmore and Sporns, 2009; Fornito et al., 2012). TMS is a non-invasive technique that induces focal electric currents through rapidly changing magnetic fields, enabling modulation of cortical excitability and large-scale network interactions. Extensive evidence demonstrates that TMS can reshape activity within prefrontal–limbic circuits implicated in depression, SCZ, and obsessive-compulsive disorder (Eldaief et al., 2013; Liston et al., 2014; Carmi et al., 2019). Further, rTMS protocols exert frequency- and pattern-dependent effects, with high-frequency stimulation typically enhancing cortical excitability and low-frequency stimulation producing inhibitory effects. These properties allow TMS to normalize hypoactive dorsolateral prefrontal regions in depression or attenuate hyperactive cognitive control and motor networks in obsessive-compulsive disorder (Fitzgerald et al., 2006; Carmi et al., 2019). Importantly, neuroimaging studies indicate that the therapeutic effects of TMS extend beyond the stimulation site, producing coordinated changes in functional connectivity within the default mode, salience, and frontoparietal networks, thereby supporting system-level reorganization rather than isolated focal modulation (Fox et al., 2014; Drysdale et al., 2017). In contrast, tDCS offers a complementary, non-invasive approach by applying weak direct electrical currents to shift neuronal resting membrane potentials and bias ongoing neural activity. Although its effects are subtler than those of TMS, tDCS has been shown to enhance synaptic plasticity, facilitate learning-related processes, and modulate connectivity within executive and affective networks (Stagg and Nitsche, 2011; Chan and Han, 2020). A key advantage of both TMS and tDCS lies in their compatibility with real-time neuroimaging and electrophysiological monitoring, enabling the development of closed-loop neuromodulatory systems. These adaptive frameworks dynamically adjust stimulation parameters based on ongoing neural states, improving precision and potentially reducing interindividual variability in treatment response (Zrenner et al., 2016; Bergmann, 2018; Cappon and Pascual-Leone, 2024). Further, DBS provides a more invasive but highly targeted method for modulating subcortical structures and network hubs implicated in treatment-resistant psychiatric and neurological disorders. Originally developed for movement disorders, DBS has been successfully repurposed for major depressive disorder, obsessive-compulsive disorder, and Tourette’s syndrome, with stimulation targets including the subgenual cingulate cortex, nucleus accumbens, ventral capsule, and ventral striatum (Khosravi, 2025). Unlike open-loop stimulation paradigms, adaptive or closed-loop DBS systems detect pathological network states in real time and deliver stimulation contingently, thereby enhancing therapeutic efficacy while minimizing adverse effects (Guidetti et al., 2025). Advances in ML, connectomics, and computational modeling increasingly inform DBS targeting strategies, allowing stimulation parameters to be personalized based on individual network architecture and disease-specific circuit dysfunction (Ruffini et al., 2009; Fox et al., 2012; Chen S.-Y. et al., 2024). Crucially, neuromodulatory interventions do not exert strictly local effects but instead propagate across anatomically and functionally connected regions, reshaping large-scale network dynamics. Both TMS and DBS have been shown to alter oscillatory synchronization and temporal coordination across distributed circuits (Eldaief et al., 2023; Neumann et al., 2023). Additionally, TMS can entrain cortical oscillations in a frequency-specific manner, enhancing theta- or gamma-band coherence depending on stimulation parameters, while DBS suppresses pathological rhythms such as excessive beta synchrony. These oscillatory effects improve interregional communication and restore information flow, providing a mechanistic link between focal stimulation and global network normalization (Eusebio and Brown, 2007; Thut et al., 2017).

At the molecular and cellular levels, accumulating evidence indicates that these network-level changes are supported by activity-dependent plasticity mechanisms. Repeated neuromodulatory stimulation can induce long-term potentiation or depression-like effects through alterations in calcium signaling, receptor trafficking, and synaptic strength, thereby recalibrating excitation-inhibition balance within affected circuits (McIntyre and Anderson, 2016; Bazzari and Parri, 2019; Yamada and Sumiyoshi, 2023). Concurrent modulation of neurotransmitter systems, including glutamatergic, GABAergic, and dopaminergic signaling that further reshapes synaptic gain, oscillatory coordination, and neuromodulator tone across interconnected regions. Sustained neuromodulatory activity also engages intracellular signaling cascades that regulate gene expression and neurotrophic pathways, including brain-derived neurotrophic factor–mediated mechanisms, supporting synaptic maintenance, structural remodeling, and long-term circuit stabilization (Giordano et al., 2017; Binns et al., 2025). Collectively, these findings position contemporary neuromodulatory interventions as multiscale therapeutic tools that couple molecular and synaptic processes to circuit-level remodeling and systems-level functional recovery. By integrating connectome-informed targeting, adaptive stimulation paradigms, and mechanistic insight across biological scales, precision neuromodulation aligns closely with emerging frameworks of network-based and personalized neuropsychiatric treatment.

### 7.2 Therapeutics at the synaptic and molecular levels

Advancements in neuromodulation are accompanied by progress in molecular neuroscience, leading to the development of a new generation of synaptic and molecular therapeutics that target the cellular foundations of psychiatric dysfunction (Kargbo, 2024; Howes et al., 2025). Traditional psychotropics have mainly focused on monoaminergic systems; however, their limited effectiveness and delayed therapeutic onset highlight the necessity for more mechanistically informed strategies. Current strategies emphasize the modulation of synaptic and intracellular signaling pathways that govern plasticity, excitatory–inhibitory balance, and neuroimmune interactions (Kavalali and Monteggia, 2025). For instance, metabotropic glutamate receptor (mGluR) modulators are being investigated as viable alternatives to NMDA antagonists, providing a means to precisely regulate glutamatergic transmission while avoiding excitotoxicity. Agents that target mGluR2/3 have shown promise in diminishing hyperglutamatergic signaling and improving cognitive and affective symptoms associated with SCZ and anxiety disorders (McKenzie et al., 2025; Olivares-Berjaga et al., 2025). Further, neurotrophic enhancers, such as agents that upregulate BDNF or activate its receptor TrkB, show potential for restoring synaptic integrity and enhancing resilience against stress-induced neuronal atrophy. Small-molecule TrkB agonists and peptides that mimic BDNF function have demonstrated the ability to increase dendritic spine density, enhance learning, and reduce depressive phenotypes in preclinical studies (Numakawa and Kajihara, 2023). Furthermore, anti-inflammatory and immunomodulatory therapies are increasingly recognized as effective adjunctive treatments in psychiatry, due to the rising acknowledgment of neuroinflammation as a significant contributor to synaptic and circuit dysfunction. Minocycline, nonsteroidal anti-inflammatory drugs, and cytokine inhibitors (e.g., IL-6 and TNF-α antagonists) have demonstrated efficacy in diminishing microglial activation, oxidative stress, and maladaptive synaptic pruning (Panizzutti et al., 2023; Du et al., 2024). Additionally, targeting mitochondrial and metabolic signaling pathways provides a complementary approach to improve neuronal energy efficiency and mitigate oxidative damage (Nunes et al., 2025). These approaches collectively advance beyond mere neurotransmitter replacement to restore the dynamic cellular environment essential for sustaining synaptic signaling and network homeostasis.

### 7.3 Circuit-guided drug development

The combination of connectomics, neuroimaging, and pharmacology has led to circuit-guided drug development, which aligns molecular specificity with network-level effects. This paradigm aims to connect molecular targets with macroscopic brain function through the utilization of connectomic biomarkers for predicting and monitoring pharmacological responses (Preller et al., 2024; Loiodice et al., 2025). fMRI and PET imaging facilitate the real-time mapping of pharmacological agents’ effects on brain connectivity, thereby enabling the identification of “circuit signatures” indicative of therapeutic efficacy. Modulation of fronto-limbic connectivity correlates with antidepressant response to selective serotonin reuptake inhibitors (SSRIs) and ketamine, whereas restoration of thalamo-cortical synchrony predicts cognitive improvement following dopaminergic treatment in SCZ (Sabaroedin et al., 2022; Saberi et al., 2025). Recent developments in molecular imaging, particularly receptor-specific PET tracers, have improved the quantification of target engagement and the correlation between receptor occupancy and alterations in network activity. This integration facilitates bidirectional translation, encompassing molecular discovery to circuit-level validation and *vice versa*, thus expediting drug development (Ferrando et al., 2025). Furthermore, computational models that integrate transcriptomic and connectomic datasets facilitate the prediction of drug-target interactions and the identification of network nodes that are most sensitive to molecular perturbation. This strategy is consistent with the concept of network pharmacology, which optimizes therapeutic efficacy by modulating interconnected pathways instead of focusing on single molecular targets (Bi et al., 2025; Li and Kar, 2025). Moreover, psychiatric disorders are fundamentally network-based, and drug development guided by circuit architecture offers a biologically coherent approach to achieving precision in treatment design.

### 7.4 Digital phenotyping and biomarker-guided clinical trials

The ultimate challenge in translational psychiatry is the integration of digital phenotyping and biomarker-guided clinical trial design. This approach utilizes advancements in wearable technology, mobile health platforms, and real-time behavioral analytics to enhance the personalization of monitoring and prediction of treatment responses (Alam et al., 2025; Linardon et al., 2025). Conventional clinical assessment instruments frequently depend on subjective symptom reporting, which is characterized by limited temporal resolution and ecological validity. Digital phenotyping employs continuous passive data collection through smartphones, wearables, and biosensors to gather behavioral, physiological, and environmental variables, including sleep patterns, speech dynamics, social activity, and movement (Jilka and Giacco, 2024). High-frequency, multimodal datasets facilitate the identification of digital biomarkers associated with neurobiological states and network-level alterations. Integrating digital measures with neuroimaging, electrophysiology, and genomic data enables researchers to develop multilayered predictive models that can identify early warning signals of relapse, monitor treatment adherence, and dynamically adjust interventions (Choo et al., 2024; Jiang et al., 2024; McGinnis et al., 2024). Alterations in speech prosody or gait dynamics may indicate disruptions in the fronto-striatal network that occur prior to the exacerbation of clinical symptoms. ML algorithms utilized on this data can categorize patient subtypes, forecast treatment responsiveness, and determine optimal therapeutic windows for intervention (Yang M. et al., 2024). Moreover, biomarker-guided clinical trials are essential for advancing precision psychiatry, as they utilize biological and digital markers for inclusion criteria, stratification, and outcome assessment. Neuroimaging biomarkers, including functional connectivity in the default mode and salience networks, function as objective endpoints for assessing treatment efficacy, whereas molecular and genetic profiles inform drug selection and dosing strategies (Preller et al., 2024; Etkin et al., 2025). Adaptive trial designs, which allow treatment protocols to evolve based on real-time biomarker feedback, improve efficiency and individualization in clinical research. A comprehensive translational framework in psychiatry highlights the importance of network-informed, precision-based treatment approaches (Kaizer et al., 2023). Neuromodulation reinstates connectivity, molecular therapeutics rectify signaling and inflammation, while circuit-guided pharmacology synchronizes interventions with network dynamics. The integration of digital phenotyping for real-time personalization with these advancements establishes precision psychiatry based on computational modeling, measurable biomarkers, and individualized neural and molecular profiles (Zhao et al., 2025). The therapeutic landscape in psychiatry is evolving from mere symptom management to a focus on reinstating the biological principles of neural communication, including synchrony, adaptability, and resilience. Translational neuroscience integrates advancements in neurotechnology, molecular biology, and data science, transforming the diagnosis, monitoring, and treatment of psychiatric disorders (Gordon et al., 2024; Yoon et al., 2024). This approach fosters a future of mental healthcare that is predictive, preventive, and personalized.

## 8 Integrative framework: towards a unified theory of neural signaling in psychiatry

A comprehensive understanding of psychiatric disorders necessitates an integrative, multiscale model that connects molecular signaling abnormalities to the cognitive, emotional, and behavioral dysfunctions characterizing clinical phenotypes (Reininghaus and Morgan, 2014). Evidence from molecular neuroscience, systems biology, and network psychiatry indicates that mental disorders are not confined to specific neurotransmitter systems or brain regions. Instead, they are distributed network diseases resulting from disruptions in the hierarchical flow of neural information (Zuo et al., 2021). Alterations in receptor function, intracellular signaling cascades, and ion channel dynamics at the molecular level disrupt synaptic plasticity and excitatory-inhibitory balance, thereby impairing the brain’s capacity to encode, predict, and integrate sensory and cognitive inputs (Imbrici et al., 2013). These perturbations ascend through neural circuits, disrupting oscillatory synchronization and large-scale network integration, ultimately resulting in disturbances in cognition, affect regulation, motivation, and perception. Further, dysregulation of critical pathways, such as NMDA receptor-mediated glutamatergic signaling, calcium-dependent second messenger systems, and neurotrophic cascades like BDNF-TrkB and PI3K-Akt-mTOR, compromises synaptic stability and adaptability (Wüstner et al., 2024; Freudenberg et al., 2025). This results in diminished LTP, maladaptive plasticity, and ineffective neural communication. The loss of molecular precision leads to disorganization at the microcircuit level, especially in cortical and limbic regions. This results in impaired coordination between excitatory pyramidal neurons and inhibitory interneurons, which generates cortical noise, diminishes information fidelity, and contributes to the abnormal inference patterns associated with psychiatric symptoms (Vinogradov et al., 2023; Caya-Bissonnette and Béïque, 2024). From a computational perspective, disruptions at the molecular and microcircuit levels result in prediction errors and inaccurate hierarchical inference, as outlined in predictive coding models, which in turn lead to distorted perceptions, beliefs, and emotional responses. At the systems level, localized disruptions escalate into extensive dysconnectivity, a defining characteristic of psychiatric disorders (Qela et al., 2025). Additionally, decreased oscillatory coherence in gamma and theta frequency bands indicates impaired synchronization between thalamo-cortical and fronto-limbic circuits, resulting in deficits in cognitive control, working memory, and emotion regulation. Graph-theoretic analyses demonstrate patterns of hub vulnerability, reduced global efficiency, and modular segregation in various disorders (Palmisano A. et al., 2024). Reduced connectivity in the default mode and executive networks leads to cognitive rigidity and rumination in depression, whereas abnormal activation of the salience network results in impaired reality testing and salience attribution in SCZ (Katayama et al., 2025). Moreover, psychiatric disorders, despite their clinical heterogeneity, exhibit a fundamental mechanism characterized by an imbalance in neural signaling. This imbalance disrupts the dynamic equilibrium among excitation and inhibition, plasticity and stability, as well as prediction and adaptation (Lányi et al., 2024). Disorder-specific variations arise from distinct molecular perturbations: NMDA receptor hypofunction and GABAergic deficits in SCZ lead to cortical disinhibition; monoaminergic and neurotrophic dysregulation in depression diminishes prefrontal-limbic communication; calcium signaling and mitochondrial dysfunction in bipolar disorder result in oscillatory instability; and aberrant synaptic pruning and hyperconnectivity in ASD disrupt global integration (Cui et al., 2024; Zhang T. et al., 2024). The distinct molecular signatures lead to common computational outcomes, specifically disrupted hierarchical inference and network-level dysconnectivity. The proposed unified theory of neural signaling views mental disorder as an emergent phenomenon resulting from recursive interactions at molecular, circuit, and network levels (Harris, 2025). A single perturbation, such as calcium dysregulation, can propagate through this hierarchy, modifying synaptic efficacy, reshaping oscillatory coupling, reorganizing network topology, and resulting in behavioral symptoms, such as cognitive inflexibility or affective instability. Therapeutic interventions, including molecular modulators, neuromodulation techniques, such as TMS and DBS, and computationally guided closed-loop systems, can restore function by recalibrating signaling precision across various levels of this hierarchy (Su et al., 2024; Dai et al., 2025; Langbein et al., 2025). This framework has significant translational implications, promoting the multimodal integration of genomic, transcriptomic, electrophysiological, and neuroimaging data to establish individualized “signaling fingerprints.” AI and computational modeling can translate these signatures into predictive models for symptom trajectories and treatment responses (Kanyal et al., 2024; Huang and Shu, 2025). This unified theory posits that psychiatric disorders, despite their clinical diversity, share a commonality in the disruption of neural communication across biological scales. Decoding this language will facilitate the transition of psychiatry from descriptive diagnosis to mechanistic, precision-based interventions aimed at restoring balance to the signaling networks that govern thought, emotion, and behavior.

## 9 Cross-scale integration of functional connectivity with molecular and cellular substrates

Recent discoveries in multimodal neuroscience give evidence to the importance of studying the relationship between functional connectivity at the large scale and its underlying molecular and cellular determinants. An example of this is the effect of changes in functional connectivity patterns of large-scale brain networks (e.g., prefrontal-limbic networks, thalamo-cortical networks, default mode networks) on network dysregulation across psychiatric conditions (i.e., disruptions in functional connectivity) (Sheline et al., 2009; Menon, 2011; Hawrylycz et al., 2012). The data now show that the patterns observed may be due to gene expression, cell-type composition, and timing of development, not merely as system-level phenomena (Hawrylycz et al., 2012). Further, integrating neuroimaging data with transcriptomic data and data focused on the specific cell types within the brain has better shown the relationship between functional connectivity patterns and underlying cellular conditions. Analyses comparing resting-state functional connectivity patterns with the profiles of gene expression demonstrated that functional connectivity patterning is influenced by the molecular gradients that determine synaptic signaling, neurotransmitter receptor expression, and the numbers and location of inhibitory interneurons (Goulas et al., 2014; Fulcher et al., 2019; Batiuk et al., 2020; Markello et al., 2022; Wang et al., 2023). In addition, analyses of developmental transcriptomic datasets show that critical windows exist for both the maturation of synapses and the balance between excitation and inhibition and that these critical windows offer a mechanistic means of explaining the different developmental trajectories associated with psychiatric disorders (Kang et al., 2011; Whitaker et al., 2016; Birnbaum and Weinberger, 2017). Moreover, dynamic functional connectivity studies bolster this framework by connecting the switch-like state-dependent network behavior with the underlying molecular and cellular substrates. Changes in connectivity dynamics have been found to correlate with changes in the expression of genes associated with synaptic plasticity, myelination and immune signaling, indicating that the instability of a network may reflect cellular stress or deficits in circuit modulation (Hansen et al., 2021; Wang et al., 2023). In SCZ, the use of multi-omics approaches, through a combination of functional connectivity, electrophysiology and transcriptomics, has revealed common abnormalities present across multiple molecular pathways, circuit motifs and systems-level organization, suggesting that dysfunction can propagate hierarchically (Fornito et al., 2019; Anderson et al., 2020; Guan et al., 2022). Likewise, a study conducted by Li W. et al. (2025) introduces a region-wise functional connectivity switching index revealing age-dependent network drivers in ASD, linking abnormal dynamics to transcriptomic signatures, neurotransmitter systems, and interneuron–astrocyte cellular mechanisms (Li W. et al., 2025).

Collectively, these studies suggest a mechanism by which disruptions at the molecular and cellular level (i.e., neurotransmitter receptor dysregulation, interneuron dysfunction, epigenetic repression of plasticity genes and neuroimmune activation) lead to biases in circuit-level communication, abnormal oscillatory coordination and reconfiguration of large-scale network topologies. By integrating this emerging literature across multiple scales, this review positions functional connectivity abnormalities within the context of biological mechanisms, moving from descriptive network phenotypes to mechanistic interpretability. This integrative approach is critical for transforming the field of network neuroscience into clinically applicable biomarkers and developing targeted precision interventions aimed at the upstream molecular contributors to systems-level dysfunction.

## 10 Conclusion and future directions

**FIGURE 7:**
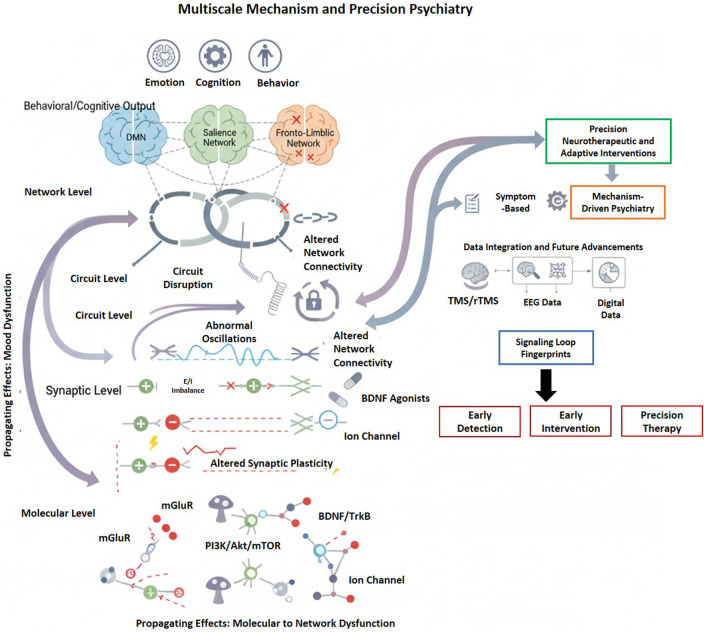
Multiscale mechanisms and precision psychiatry: Molecular and synaptic disruptions, including mGluR and BDNF/TrkB signaling imbalances, ion channel dysfunction, and altered excitatory/inhibitory (E/I) ratios, lead to circuit-level abnormalities that result in aberrant oscillations and compromised network connectivity within the default mode, salience, and fronto-limbic networks. These network-level disruptions present as emotional, cognitive, and behavioral impairments. Further, integrative, data-driven methodologies utilizing fMRI, EEG, connectomics, and digital phenotyping enhance mechanism-driven psychiatry. Additionally, advanced neurotherapeutic approaches, such as TMS/rTMS, BDNF agonists, and precision neuromodulation, provide early identification, intervention, and individualized therapy, addressing disturbances across several levels from molecular processes to behavioral results.

The current review highlights that psychiatric disorders are multiscale disruptions of neural communication, involving dynamic interactions among molecular, synaptic, circuit, and network-level dysfunctions that lead to the cognitive, emotional, and behavioral impairments associated with mental disorders. Mechanistic insights at various levels indicate that disruptions in neurotransmission, receptor signaling, intracellular pathways, and ion channel dynamics converge on a common theme of compromised excitatory-inhibitory balance and synaptic plasticity. Further, molecular perturbations propagate through neural circuits, resulting in dysregulated oscillations, disrupted network topology, and altered connectivity within core systems, including the DMN, salience networks, and fronto-limbic networks. This framework integrates molecular psychiatry, systems neuroscience, and computational modeling to conceptualize psychiatric pathology as an emergent property of hierarchical signaling dysregulation, rather than as discrete neurotransmitter deficits. This paradigm has prompted a transition from symptom-based interventions to mechanism-guided strategies focused on restoring signaling precision and network integrity. Additionally, precision neuromodulation techniques, such as TMS, DBS, and closed-loop systems, facilitate circuit-specific modulation. Concurrently, molecular and synaptic therapeutics that target mGluR, BDNF-TrkB, and PI3K-Akt-mTOR pathways seek to restore cellular homeostasis. Moreover, AI-driven computational models and multimodal connectomics are redefining diagnostic boundaries by integrating molecular, electrophysiological, and behavioral data to create individualized “signaling fingerprints” for mechanistic classification and targeted therapy. Current approaches, despite advancements, encounter significant limitations, notably the difficulty of distinguishing causality from correlation in human neuroimaging and omics data. The majority of evidence is associative, highlighting translational gaps between molecular perturbations in experimental models and complex behavioral outcomes in clinical populations. The temporal evolution of signaling dysfunction throughout development and disease progression is inadequately understood, hindering early detection and preventive strategies. Future research should focus on longitudinal, multimodal studies that combine genomic, epigenetic, neurophysiological, and behavioral data to delineate causal pathways of neural dysfunction. Single-cell and spatial omics technologies are promising for elucidating cell-type-specific and regionally localized signaling changes, thereby connecting molecular pathology with systems-level connectivity. The integration of AI-based mechanistic inference frameworks, including graph neural networks, causal modeling, and dynamic Bayesian learning, enables the inference of directionality in molecular-to-network interactions and the prediction of treatment responses with high precision. Thus, future initiatives must emphasize cross-species translational pipelines that connect mechanistic insights from model systems to human data via shared connectomic and transcriptomic signatures. Embedding digital phenotyping and real-time biosensing into clinical trials facilitates continuous monitoring of neural and behavioral states, thereby advancing adaptive, biomarker-guided interventions. The integration of molecular biology, computational neuroscience, and AI signifies a pivotal advancement in psychiatry, wherein diagnosis and treatment are based on the principles of neural signaling and network organization. Integrating causal modeling with longitudinal, multiscale data enables the field to progress from correlation to a predictive, mechanistic, and precision-based psychiatry, which aims to restore the harmony of neural communication underlying cognition, emotion, and behavior (Figure 7).

## Glossary

SCZ: schizophrenia
MDD: Major Depressive Disorder
PTSD: Post-traumatic stress disorder
ASD: Autism spectrum disorders
AI: Artificial Intelligence
NMDA: N-methyl-D-aspartate
AMPA: 3-hydroxy-5-methyl-4-isoxazolepropionic acid
PV: Parvalbumin-Positive
LTP: Long-Term Potentiation
LTD: Long-Term Depression
PI3K: Phosphoinositide 3-Kinase
Akt: Protein Kinase B
mTOR: Mammalian Target of Rapamycin
MAPK: Mitogen-Activated Protein Kinase
ERK: Extracellular Signal-Regulated Kinases
BDNF: Brain-Derived Neurotrophic Factor
TrkB: Tropomyosin Receptor Kinase B
Na+: Sodium
K+: Potassium
Ca2+: Calcium
Cl-: Chloride
CNS: Central Nervous System
IL-1β: Interleukin-1β
IL-6: Interleukin-6
TNF-α: Tumor Necrosis Factor-Alpha
BBB: Blood-Brain Barrier
HPA: Hypothalamic-Pituitary-Adrenal
ROS: Reactive Oxygen Species
mtDNA: Mitochondrial DNA
AMPK: AMP-Activated Protein Kinase
PGC-1α: Peroxisome Proliferator-Activated Receptor Gamma Coactivator 1-Alpha
TMS: Transcranial Magnetic Stimulation
DBS: Deep Brain Stimulation
CSTC: Cortico-Striatal-Thalamo-Cortical
OCD: Obsessive-Compulsive Disorder
vmPFC: Ventromedial Prefrontal Cortex
DMN: Default Mode Network
ML: Machine Learning
GWAS: Genome-Wide Association Studies
miRNA: microRNA
CpG: Cytosine-Phosphate-Guanine
HDACs: Histone Deacetylases
fMRI: Functional Magnetic Resonance Imaging
EEG: Electroencephalography
PET: Positron Emission Tomography
CNNs: Convolutional Neural Networks
GNNs: Graph Neural Networks
tDCS: Transcranial Direct Current Stimulation
mGluR: Metabotropic Glutamate Receptor
